# Mechanical Joining of Fibre Reinforced Polymer Composites to Metals—A Review. Part I: Bolted Joining

**DOI:** 10.3390/polym12102252

**Published:** 2020-09-30

**Authors:** Anna Galińska

**Affiliations:** Faculty of Power and Aeronautical Engineering, Institute of Aeronautics and Applied Mechanics, Warsaw University of Technology, Nowowiejska 24, 00-665 Warsaw, Poland; agalinska@meil.pw.edu.pl

**Keywords:** fibre reinforced plastic composite, bolted joining, bearing strength, delamination

## Abstract

As the fibre reinforced plastic composites gain larger and larger share in industry, the problem of joining them with metal elements becomes significant. The current paper is the first part of the literature review, which gathers and evaluates knowledge about methods suitable for mechanical joining of composite and metal elements. This paper concerns bolted joining, because this method of mechanical joining is widely used for joining composite materials. The paper describes failure modes of bolted joints in composite materials, the influence of the bolt clamping torque, the clearance between the bolt and the hole and aging on the performance of the joint, drilling techniques used in composite materials in order to minimize damages, different fastener types, inspection techniques, and finally, the techniques that have been developed in order to improve the strength of the bolted joints in composites. Since the hole drilled in a composite material in order to perform bolted joining is a weak point of the structure, those techniques: bonded inserts, titanium foil internal inserts, fibre steering, additional reinforcement, and moulded holes, mainly aim to improve the strength of the hole in the composite. The techniques have been discussed in details and compared with each other in the summary section.

## 1. Introduction

Fibre reinforced composite materials gain progressively larger share in the structures of modern aircraft. The amounts of composite laminates used in the Boeing 787 Dreamliner and Airbus A350 exceed 50% of the vehicle weight [1,2]. Even greater share of composite materials can be found in modern yachts [3,4,5]. Composite materials have been also more and more frequent in the automotive industry [6,7] since 1981, when Mc Laren built the first carbon composite monocoque for the F-1 car. Currently composite materials are proposed even for emergency bridges and other civil engineering objects [8,9,10,11,12]. Since the metal elements are and will be indispensable in these structures, the problem of joining composite and metal elements becomes more and more important as the share of composite elements in the structures increases. The joint used in a composite structure is usually the weakest point of the structure, and thus determines the structural efficiency [13]. The most popular ways of joining composite parts are bolted joining and adhesive bonding [14,15,16]. The bolted joining has many advantages over adhesive bonding [14,15,16,17,18,19,20]:

- the ease of assembly/disassembly,
- the ease of part replacement,
- the ease of repair,
- the ease of inspection,
- the ease of airworthiness certification,
- tolerance to environmental effects.

Moreover, the choice of an adhesive which works properly with both composite and metal elements and the proper way of surface treatment for adhesive joining is a demanding and tedious task [21,22,23]. Therefore, the bolted joints are still used despite several drawbacks of this joining method in composite materials. The drawbacks of the bolted joining are:

- the stress concentrations caused by the fastener hole,
- the disruption of the fibre continuity during drilling, which severely reduces the load carrying capacity [24,25,26],
- undesirable damages (such as delamination, fibre pull-out, and microbuckling [26]) induced by drilling the holes which drastically reduce strength against fatigue, thus degrading the long-term performance of composite laminates [27].

In consequence, the bolted joint effectiveness in composites is worse than in metals, which detract from the weight advantage of composites [28,29]. Apart from the bolted joining, alternative methods of mechanical joining composite materials to metals have been developed. Those methods include:

- riveting,
- cinching,
- form-locked joints,
- pin joints,
- loop joints.

The number and complexity of mechanical methods of joining composite and metal materials makes the choice of the proper method tedious and difficult. Thus, the current paper along with an accompanying paper [30] provides the literature review which describes the level of development, advantages, and disadvantages of methods designed for joining composite and metal materials. Recent review work on mechanical joining fibre reinforced plastic (FRP) composites to metal is moderate. The most recent review work concerning the bolted joints in composite materials by Thoppul et al. focuses mainly on the mechanics and mechanical testing of bolted joints in polymer-matrix composite structures [14]. There are also several works reviewing various methods of joining composite-composite and composite-metal structures [31,32,33,34,35], but none of them focuses solely on mechanical joining and covers all the joining methods developed so far. Therefore, the current works present a review which fulfils both those conditions. This part of the review devoted to bolted joining reviews:

- modes of failure of bolted joints focusing on the mechanisms characteristic for composite materials,
- the influence of the bolt clamping torque, the clearance between the bolt and the hole and aging on the performance of the joint,
- drilling techniques used in composite materials in order to minimize damages,
- different fastener types,
- inspection methods,
- techniques that have been developed in order to improve the strength of bolted joints in composites.

Since a hole drilled in a composite material in order to perform bolted joining is a weak point of the structure, those techniques:

- bonded inserts,
- titanium foil internal inserts,
- fibre steering,
- additional reinforcement,
- moulded holes

mainly aim to improve the strength of the hole in the composite. As this last part is particularly interesting from the point of view of a designer looking for new solutions to enhance the performance of composite structures, those techniques have been discussed in details and compared with each other in the summary section.

## 2. Modes of Failure

Although the failure modes of bolted joints are similar in metal and composite materials, the phenomena occurring during the failure are different. The joints in metals are known to outperform the joints in composites [28,29]. According to Duthinh the better performance of bolted joints in metals may be credited to the ductility which relieves the stress concentrations. Such ductile behaviour does not exist for FRP composite, because each of its constituents (the matrix and the fibres) behaves in a brittle manner up to the failure [29,36,37,38]. On the other hand, subcritical damage modes such as

- transverse matrix cracks,
- axial splits (fibre/matrix shearing),
- delaminations between adjacent layers around the highly stressed regions of the hole edgecontribute to that stress concentration relief in composites [37].

**Figure 1:**
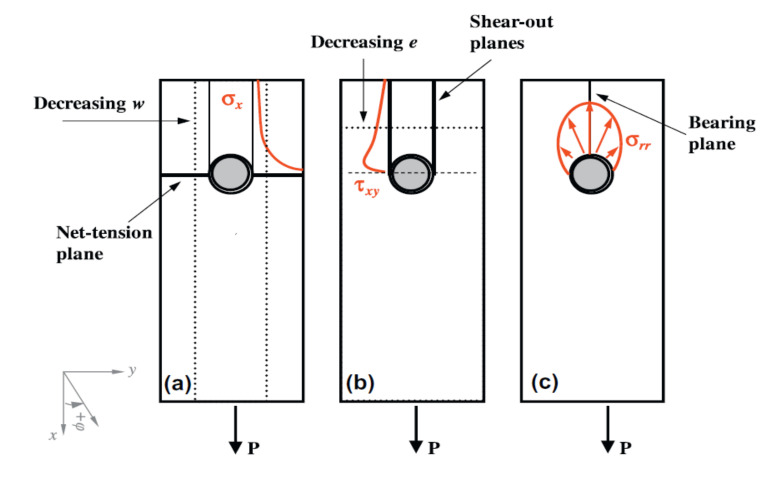
Modes of bolted joint failures: (**a**) net tension failure, (**b**) shear-out failure, and (**c**) bearing along with critical stress distribution [37].

The basic failure modes in bolted fibre reinforced materials are [14,17,24,37] (Figure 1):

- bearing,
- net-tension,
- shear-out.

In addition, out-of-plane loading can cause partial or complete pull-out of the bolt from the composite structure [14]. Typical critical stress distributions around a pin-loaded hole are

- the normal stresses at the net-tension plane,
- the shear stresses at the shear-out plane,
- the radial bearing stresses at the bearing plane

as illustrated in Figure 1 [37]. The net-tension failure is associated with matrix and fibre tension failures due to stress at the hole edge [14]. Shear-out and bearing failures result primarily from the shear and compression failures of fibres and matrix [14]. The tension and shear stresses decay rapidly away from the hole edge at the net-tension and shear-out planes (Figure 1a,b). Maximum radial compression (bearing) stress develops at the bearing plane due to the fastener/hole interaction [37]. Net-tension failure occurs when the width to diameter ratio is small or when the ratio of by-pass load to bearing load is high [14,37]. Shear-out failure mode is observed in laminates with adequate width against the net-tension mode, but insufficient free edge distance to relieve the high shear stresses developed at the shear-out plane [37]. Bearing mode is caused by the compressive stresses and includes matrix cracking, fibre microbuckling, and kinking with a significant amount of delamination [37]. From these failure modes only bearing damage produces a progressive failure, indicated by the non-linear behaviour of the joint, thus composite bolted joints are usually designed to fail under this mode [14,17,24]. The other two failure modes may suddenly reduce the load carrying capacity or cause instant failure of the whole structure [24].

**Figure 2:**
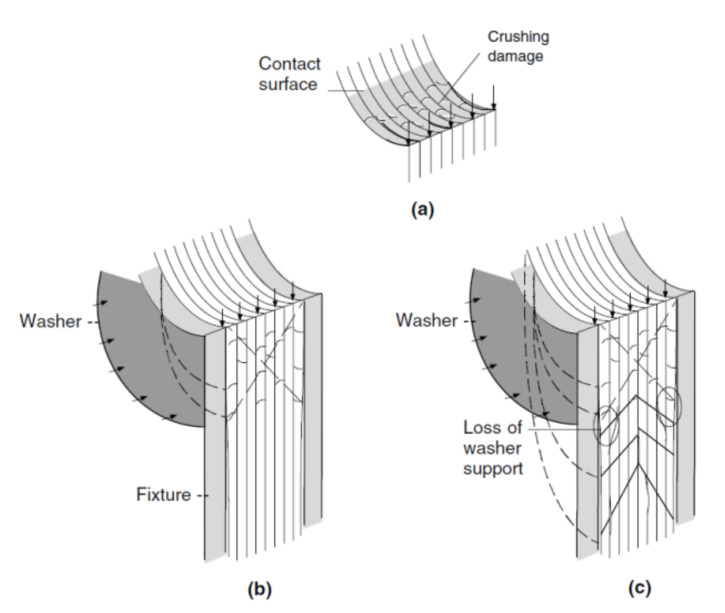
Schematic presentation of the bearing failure mechanisms: (**a**) crushing damage, (**b**) damage within lateral constraint (inside-washer region), and (**c**) damage without lateral constraint (outside-washer region) [39].

The bearing failure is also the most complicated failure mode in the case of composite laminates. Fibre micro-buckling, matrix cracks, delamination, and shear caused by the compressive load imposed by the bolt result in bearing failure of the laminate [39,40]. Figure 2 presents the phenomena occurring in composite during bearing failure of a bolted joint with washers observed on SEM pictures [39].

The joint strength depends on different parameters, such as [17]:

- joint configuration,
- bolt torque,
- laminate and bolt materials,
- laminate lay-up,
- temperature.

The type of matrix in composite also affects the strength. Two material systems were selected for a comparison of bearing strength in the work by Xiao and Ishikawa: polyimide and epoxy graphite reinforced composite laminates with quasi-isotropic lay-up [39]. X-ray photographs have shown that the epoxy–matrix laminate is more vulnerable to matrix cracking and delaminations induced by bearing than polyimide-matrix laminate [39]. The failure mode and the failure strength depend also on the stacking sequence [13]. For example the static net-tension strength of the bolted joints in the [±60/0_8_]_S_ and [±45/0_8_]_S_ glass-epoxy laminates made of UD tapes is about 20% higher than that of the bolted joints in the [90_2_/0_8_]_S_ laminate [13]. As environmental conditions, temperature and moisture, have also significant influence on the behaviour of bolted joints in polymer-matrix composites—the strength of the joints subjected to a combination of hot/wet conditions have been found to be significantly lower than of those tested in ambient conditions [14,41]. Turvey and Wang carried out an experimental study of the effects of hot/wet conditions on the load carrying capacity of pultruded glass reinforced plastic single-bolt joints. It was found that more than 60% of the joints tension strength was lost after being immersed in water for 6.5 weeks at 60 °C [41]. Moreover, failure modes of single-bolt tension joints also varied depending on the temperature and water immersion period. For example, the specimens that were designed to fail under net-tension mode, failed under bearing failure with increasing temperature [14,41].

## 3. Clamping Torque

In bolted joint laminates, high stress concentrations appear near the fastener holes. However, if the substrates are subjected to clamping torque, which causes frictional force between the washers and the composite laminate, the washers share a portion of the external load in the vicinity of the washer area and the friction force releases the concentrated stress surrounding the hole-edge [25]. The stress is thereby redistributed, which could reduce stress concentrations, resulting in a higher failure load [25]. This dependence has been investigated in several works. Thoppul et al. and Kapi et al. demonstrated the positive effect of the friction force that leads to increased bearing strength [14,42]. For carbon/epoxy laminates bearing strength grows when the clamping pressure is increased. On the other hand, the negative effect of the clamping force is that out-of-plane stresses can lead to a premature failure of the joint, because the through-the-thickness properties of fibre reinforced composites are very poor and thus the composite is vulnerable to damage and failure in this direction [14,43]. In order to understand the effects of clamping force on a bolted joint, Choi et al. used two values of clamping torque: 30 Nm and 50 Nm [25]. Unidirectional E-glass/epoxy composite and carbon/epoxy composite were used to produce laminates with stacking sequence of ±45°. The results show that the higher clamping torques led to the higher failure loads. For the E-glass/epoxy composites, the failure load increased by 16.3% when the clamping torque changed from 30 Nm to 50 Nm. Similarly, the failure load for the carbon/epoxy composite increased by 14% for the same increase of the clamping torque [25]. Park investigated the influence of the clamping force on the bearing strength of bolted joint in carbon/epoxy quasi-isotropic composite [44]. The clamping pressures were the following: 0, 5.2, 29.3, and 58.6 MPa. The results show great increase in the ultimate bearing strength when the clamping pressure is increased from 0 to 5.2 MPa (over 100%) and moderate strength increase when the clamping pressure is increased further. After applying clamping pressure the failure mode changes from a catastrophic to a progressive one [44]. Khashaba et al. investigated the effects of tightening torque and washer outer diameter size on the strength of bolted joint in glass/epoxy composite materials [45]. The strength of bolted joints with various values of tightening torque (0 Nm, 5 Nm, 10 Nm, and 15 Nm) and washer sizes (outer diameter of washers 14 mm, 18 mm, 22 mm, and 27 mm) was determined in tension tests. The highest bearing strength was achieved for 15 Nm torque and 18 mm washer diameter [45]. Both increasing the torque and decreasing the washer diameter increases the contact pressure. The results indicate that, for glass/epoxy composite, increasing the contact pressure increases joint bearing strength, but only to some extent, until the pressure is so large that it causes composite cracking. The results obtained by Chishti et al. for carbon reinforced composite show that the application of 2.1 Nm of bolt torque led to a significant increase in bearing stress (33%) compared to the finger-tight case, whilst only a minor increase (3%) in the ultimate failure stress [15]. Doubling the amount of bolt torque, however, did not show any further improvement, with only marginal changes in the bearing stress and ultimate stress [15].

## 4. Clearance

**Figure 3:**
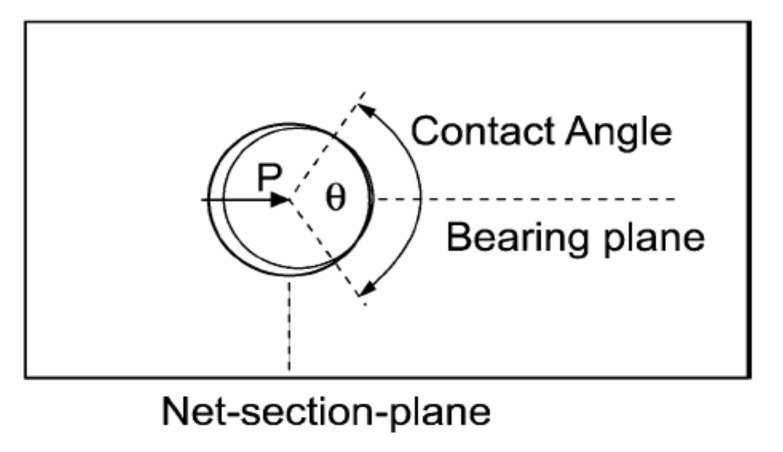
Contact angle for bolt-hole bearing [47].

The higher the clearance between the bolt and the hole, the lower the contact area between them is [46,47] (Figure 3). The decrease of the contact area causes higher bearing stresses and in result, lower bearing strength.

Therefore, the clearances between the bolt and the hole should be kept as low as possible, in order to assure the highest possible bearing strength of the joint. Mechanically fastened joints commonly adopted in aerospace structures are known to have tight geometric tolerances of both the fasteners and the machined holes. However, if composite materials are to be used in mass production, different tolerance levels may be necessary for the joining of components to allow shorter manufacturing times and minimise production costs [47]. The certain level of clearances is, therefore, unavoidable in the composite bolted joints. The influence of the clearance in the bolted joint has been investigated in several works. Kelly and Hallström investigated three different clearance levels [47]. The specimens were machined from carbon fibre/epoxy laminates. Two different laminate thicknesses with stacking sequences [0/45/90/−45]_S_ and [0/45/90/−45]_S2_ and two clearance values 1.55% and 3.05% of the nominal hole diameter were investigated. The bearing strength at 4% hole deformation was shown to decrease down by 19% for the thinner laminate and by 6% for the thicker laminate as the clearance was increased to 3.05%. However, the ultimate bearing strength remained almost constant and independent of the clearance between the bolt and the hole. The application of lateral clamping load was found to increase the bearing values of the laminates by approximately 20% in comparison to laminate loaded without any clamping force [47]. Zhai et al. investigated three different torque levels: 0.5 Nm (‘‘finger tight’’ condition), 2.1 Nm and 4.2 Nm, and clearences: neat-fit, 40, 80 and 120 μm for a 4 mm nominal diameter of hole in the case of single-lap, countersunk composite joints [46]. It has been found that the bolt-hole clearance reduces the joint stiffness and strength and that increasing bolt torque from 0.5 Nm to 4.2 Nm increases the bearing strength by 6.7%, 7%, 4.2%, and 5.9% for 0, 40, 80, and 120 μm clearance, respectively [46]. McCarthy et al. investigated the influence of the clearances on the bearing strength of carbon/epoxy bolted joint specimens [28]. The clearances chosen for this study were neat-fit, 80, 160, and 240 µm for a nominal bolt diameter 8 mm [28]. The trend of slight decrease in the bearing strength was visible for the bolts with protruding heads in quasi-isotropic laminate. The strength decreased by 16% as the clearance increased from 0 to 240 μm. However, no such evident trend was observed for countersunk bolts [28]. Chishti et al. also showed that the introduction of 220 μm of clearance for 6.35 mm bolt diameter led to a significant (17%) reduction in the bearing stress in carbon/epoxy composite, though only a slight reduction in the ultimate stress [15]. Doubling the clearance only reduced the bearing stress by 7%, with a further slight reduction in ultimate stress [15]. An opposite effect of the bolt clearance on the fatigue strength of bolted joints was described by Wei et al. [48]. An experimental investigation was conducted on double lap-type single bolted composite joints. The tension–compression test was selected to evaluate the fatigue tests. The joints with blind bolts with four clearances: 0% (neat fit), 0.5%, 1.8%, and 3% were tested separately. The appropriate levels of fatigue stress were determined by the ultimate bearing strength obtained from the static tensile tests. The experimental results show that the clearance can improve fatigue life of bolted joints compared to neat fit [48]. For the composite material studied in this paper, the joints with 3% clearance have the best fatigue performance for lower bearing stress (less than 540 MPa); however, for higher bearing stress (more than 660 MPa), the best clearance is 1.8% [49]. It was not investigated why the clearance improves the fatigue performance.

## 5. Aging

Generally the performance of fibre reinforced polymer composites deteriorates with the time of exposure to environmental conditions such as

- moisture,
- elevated temperature,
- UV radiation.

Elevated temperatures affect the mechanical properties of the FRP composites leading eventually to thermal degradation of the thermoset polymer matrix if the temperature exceeds its glass transition temperature. However, even temperatures far below the glass transition temperature lead to deterioration of bolted joint performance in polymer composites. Turvey and Wang conducted tension tests of pin loaded glass fibre reinforced plastic (GFRP) composite at three temperatures 20 °C, 60 °C, and 80 °C [41]. The specimens designed to achieve bearing exhibited a reduction of 38.7% in strength at 60 °C and a reduction of 51.0% at 80 °C compared to the room temperature. In the case of the specimens designed to fail in room temperature by net-tension failure, the loss of strength in elevated temperature was even more severe: 48.5% at 60 °C and 56.4% at 80 °C and combined with the change of the failure mode from net-tension to bearing failure [41]. The strength reduction of double-lap bolted joints in GFRP composite in a wider range of temperatures: from room temperature up to 220 °C was investigated by Wu et al. [50]. The strength of the joints was reduced compared to the strength in the room temperature by 14% at 60 °C, 38% at 100 °C, 64% at 140 °C, 78% at 180 °C, and 85% at 220 °C [50]. Anwar investigated bearing strength in pin joints in GFRP composites at room temperature and at 60 °C [51]. About 44% reduction in bearing strength in elevated temperature was noticed compared to the tests at room temperature [51].

Moisture absorption, especially in elevated temperature, causes degradation of composites. Physical degradation includes plasticization and swelling of matrix [52,53,54], while chemical degradation consists of matrix hydrolysis, interface hydrolysis, and fiber degradation [54,55]. In most cases, the deterioration of a composite material during wet aging depends on temperature, hygrometric rate, and the nature of the composite. The transport of water can be facilitated by [56]:

- diffusion inside the matrix,
- imperfections within the matrix created during the fabrication (micro space, pores or cracks),
- capillarity along the fiber/matrix interface.

Turvey and Wang investigated the influence of moisture absorption on the strength of pin loaded GFRP composite [41]. The specimens had been immersed in water at room, 60 °C, and 80 °C temperature for 91 days. The immersion at room temperature brought about hardly any change for the specimens which failed by bearing and only 10% reduction in strength for the specimens failing by net-tension. More significant strength reduction was observed for the immersion at higher temperatures compared to specimens tested in elevated temperatures, but before the immersion. The bearing strength of the specimens subjected to the immersion in water in 80 °C was reduced to only 22.5% of the strength before the immersion and the net-tension strength was reduced to 30.6% [41]. Turvey and Wang surveyed also pin loaded GFRP composite which had been immersed in water for 45 days at room and 60 °C temperature [57]. The most severe strength reduction was by 28% after the immersion in room temperature and by 56% after the immersion in 60 °C [57]. Soykok et al. also investigated the strength of single lap shear glass/epoxy specimens with two bolts in a vertical row after aging in water at temperatures of 50 °C, 70 °C, and 90 °C for 1 and 2 weeks periods prior to the joint assembly and testing [54]. The bearing strength decreased gradually with the immersion time and temperature reaching a reduction of 39.5% for two-week immersion in 90 °C compared to unaged specimens. Besides that, it was found out that the joints with fasteners preloaded with 6 Nm tightening torque even after immersion offer noticeable advantage in terms of stiffness and strength compared with the finger-tightened ones [54]. Excessive immersing temperatures 70 °C and 90 °C caused mechanically fastened composite joints to fail abruptly in net-tension failure mode due to moisture induced degradation in material, whereas the joints of unaged laminates and those aged at 50 °C exhibit safe bearing failure mode [54].

As FRP composites are often used in marine applications, including yachts, hydroplanes, and flying boats, the investigation of sea water influence on bolted joints in such composites is also worth investigation. Ozen and Sayman studied bearing strength of glass-epoxy FRP specimens with two pinned holes after immersion in sea water for 24 h [58]. Some of the pins were unloaded, but the others were loaded by 3 Nm and 6 Nm torque. A drop in strength to 90% was observed after the immersion in specimens without torque preload, but virtually no drop was noticed in the specimens in which the joint was preloaded by both torque values [58]. Calabrese et al. studied the behaviour of glass/epoxy composite subjected to bearing test after exposure to salt-spraying, foggy conditions for 30 and 60 days to evaluate the induced change on the failure mechanisms and the consequent reduction of the joint mechanical performances compared to unaged specimens [59]. It was found out that the conditioning induced moderate reduction by maximum 28% of bearing strength for hole diameter of 8 mm and edge distance of 14 mm. Additionally, the conditioning caused no change in the failure mode [59].

Ultraviolet (UV) light is believed to have negative effects on most polymers, since it is associated with wavelengths of 290–400 nm and can dissociate the molecular bonds in most polymers [60]. Several works have investigated the negative effect of UV aging on the strength of polymer composites. Correia et al. [61] investigated glass/polyester composite exposed to UV radiation. A slight drop in flexural strength was observed in specimens after 84 days of exposure to radiation [61]. Nguyen et al. investigated steel/carbon fibre reinforced polymer (CFRP) composites double-strap bonded joints exposed to UV radiation for 16 days [62]. A considerable drop of the joint strength, 50% in the worst case was noticed. However, the deterioration of the performance of the joints was attributed mainly to the elevated temperature rather than by UV radiation [62]. These results are consistent with the general knowledge of the influence of UV radiation on FRP composites, which is known to affect a limited thickness near the surface of composite [60,63]. No research works focused directly on the influence of the UV radiation on the performance of bolted joints in polymer composites has been performed yet. Therefore, the influence of UV aging on FRP composites should be investigated in the future.

## 6. Fatigue

**Figure 4:**
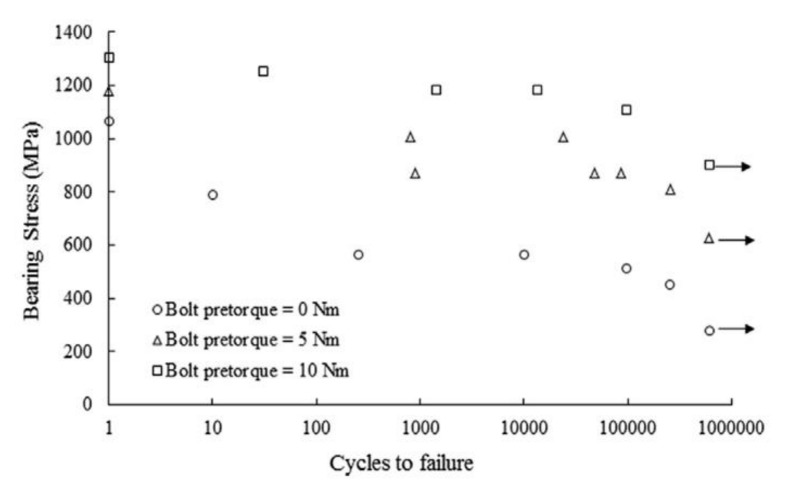
S–N graph at various bolt torque pre-tightening levels [64].

Another factor which can affect the strength of bolted joints in FRP composites is fatigue. Several experimental works have been focused on this issue. Giannopoulos et al. presented the results of static and fatigue bearing tests with different values of tightening torque [64]. The specimens were manufactured from carbon/epoxy prepreg UD tape with a quasi-isotropic lay-up. In fatigue tests, the specimen run-out was marked at 600,000 cycles. Bearing stress versus fatigue life for the specimen tested is presented in the S–N graph in Figure 4.

The residual bearing strength of the specimens rises significantly with the tightening torque, however, during static and fatigue testing at low clamp up torque, hole elongation increased relatively gradually, where high clamp up torque caused a very abrupt hole elongation increase. This may be turned into an advantage, because low torqued specimen provided warning before failure, which could potentially be utilized by structural health monitoring systems [64]. In the experimental work by Mariam et al. tensile-shear fatigue tests were performed using single lap joint specimens at the stress ratio R = 0.1 to determine the fatigue behaviour of the joints [65]. Subsequently, S–N curves were drawn for different levels of stress amplitude. The fatigue life decreased rapidly with the stress level. For the specimens with two GFRP adherends, the specimens withstood 15.1 × 10^3^ cycles for 30% of maximum fatigue load, 8 × 10^3^ cycles for 40%, 6.5 × 10^3^ cycles for 50%, 3.8 × 10^3^ cycles for 60%, 2 × 10^3^ cycles for 70%, 1 × 10^3^ cycles for 80%, and 0.6 × 10^3^ cycles for 90% [65]. Some works also investigated qualitatively the damages induced in carbon fibre reinforced plastic (CFRP) bolted joint composites. In a study by Kapidzic et al., two-bolt, double-lap joints with quasi-isotropic carbon/epoxy composite specimens were subjected to uniaxial and biaxial cyclic loading at 90 °C [66]. The constant amplitude load was applied in force control with a sinusoidal wave shape, but the maximum applied cyclic load was well below the static load failure. Therefore, all the damages were attributed to fatigue. A microscopy study of the bearing plane revealed that the main fatigue driving mechanisms were matrix cracking and fibre–matrix debonding [66]. Smith and Pascoe [67] found out that the damage induced in 0/90° ply CFRP composite with bolted induced by fatigue were:

- shear cracking in 0° plies,
- tensile cracking in 90° plies,
- interlaminar cracking.

Kinking, matrix shear cracking, and delaminations in the bearing plane were also observed in CFRP bolted composite subjected to cyclic loading and non-elastic elongation of the hole was observed [68].

Zhang et al. investigated the combined effects of seawater ageing and fatigue loading on the bearing performance and failure mechanism of CFRP/CFRP single-lap bolted joints [69]. The bolted joints were immersed in artificial seawater (3.5% NaCl solution) in 50 °C for 7 months and fatigue loads were applied to the unaged and aged joints followed by static bearing tests [69]. In the fatigue test, the loading frequency was 10 Hz and the stress ratio (minimum stress/maximum stress) was 0.1. The peak value of cyclic load was set as 10 kN, which is about 65% of the ultimate bearing load of unaged joints. After 1.0 × 10^5^ cycles of loading, the joints were tested by quasi-static load to obtain the residual bearing capacity of joints. The experimental results have shown that the aging effect caused exponential deterioration of ultimate bearing load of specimens, whereas, the combined effect of seawater ageing and fatigue loading caused a linear decrease. The failure mechanism of aged joints was mainly the shearing fracture in bearing zones; however, it changed into the delamination in bearing zones after fatigue loading [69]. A surprising finding of the research was that compared with the joints of seawater ageing alone, the bearing stiffness and capacity of joints treated by seawater ageing and fatigue loading showed a significant improvement. The possible reason is that the fretting friction occurred at the overlap surfaces during fatigue loading, which made the roughness of overlap surfaces increase and then bearing capacity increase [69]. However, this improvement was weakened with the increase of ageing time, which was about 29.2%, 26.9%, 22.6%, and 22.5% after 0, 1, 4, and 7 months of ageing, respectively [69].

## 7. Viscoelastic Effects

**Figure 5:**
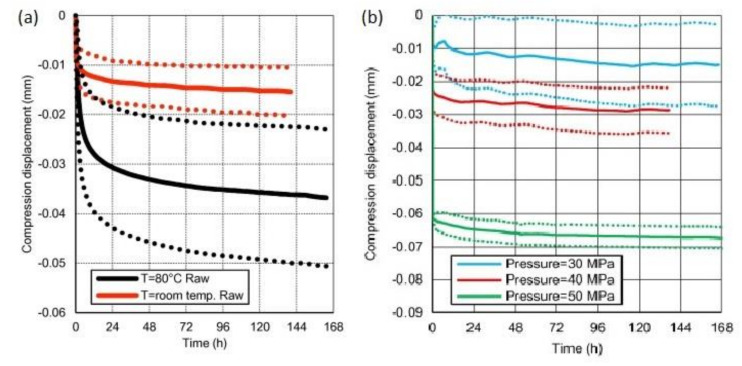
Through-the-thickness compression displacement in carbon/epoxy composite with time: (**a**) influence of temperature at pressure of 40 MPa, (**b**) influence of pressure at 25 °C temperature [70].

Polymers often exhibit creep, relaxation and other manifestations of viscoelastic behaviour, especially at elevated temperature and moisture levels [14]. Those phenomena may affect the behaviour of bolted joints in composites. The viscoelastic phenomena are particularly apparent in the through-the-thickness direction, so they may influence the initial preload of the bolt [14,70]. As some preload of the bolt in the axial direction has positive effect on the joint strength [44,70], its decrease in time induced by viscoelastic phenomena may have negative consequences for the joint strength. Therefore, this problem was studied in several works. Thoppul et al. studied the effects of various bolt preloads and external applied static and dynamic loads on the bolted load relaxation in a carbon/epoxy composite bolted joint [71]. Relaxation of 1.25–4.25% over a period of 30 h was observed depending on the initial preload and applied external loads. It was also observed that for any magnitude of external load the bolt load relaxation decreases with increasing initial preload [71]. In work by Finck et al., twenty-four specimens made of chopped roving carbon/epoxy composite were stacked, resulting in a 86.4-mm high stack [72]. The stack was placed between pressure discs of a Zwick Roell 50 kN universal testing machine, equipped with an oven chamber. A screw assembly with a force measuring washer was set up to measure the loss of preload force in time at a real setup. The force measuring washer contained strain gauges, which are used to measure the applied compression force. The screw assemblies were stored without any preload force in an oven at 120 °C for 12 h to ensure a constant temperature distribution. Afterwards, the screws were tightened to 10 kN preload relatively fast (around 1 min) manually from outside of the oven. The preloaded force was measured in time and approximately 50% loss was obtained in 360,000 s (100 h) due to the creep of the composite. Then, the gradient of the load reduction was significantly reduced [72]. Scattina et al. also studied the through-the-thickness creep in carbon/epoxy textile composite induced by compression pressure. The investigated laminate was 6.6-mm thick. The specimens were compressed by different levels of pressure and the decrease in thickness as the indicator of the creep behaviour was measured in time at room temperature or at 80 °C and the induced displacement was measured in time [70]. The main part of the total compression relaxation was reached within the first twenty four hours. Then, the displacement exhibits more moderate increase that seems governed by a linear equation [70]. The influence of the temperature is presented in Figure 5a, where the compression displacement curves at the two different temperatures and applied pressure of 40 MPa, are presented. The influence of the applied pressure is presented in Figure 5b, where the compression of displacement curves for three pressures obtained at room temperature are shown.

It can be seen that the temperature increases the intensity of creep. In the case of pressure, the data are more scattered, but, as expected, the tendency is to have a higher compression displacement with higher pressure. The trend is not linear—the intensity of the creep seems to increase with increasing pressure. This trend was confirmed also in different temperatures [70].

Relaxation in bolted thermoplastic composite joints was tested by Horn and Schmidt [73,74]. Two graphite/thermoplastic composite materials were tested with matrix made of Dupont’s IM6/KIII and ICI-Fiberite’s IM8/APC(HTA). Single shear joint specimens with both protruding head and countersink titanium fasteners were tested. Four different torque levels were applied from 0 up to 11.3 Nm. Static strength tests revealed that bearing strength of both materials was increased by as much as 28% with increasing torque. Second set of specimens was used for relaxation tests in room (25.6 °C) and elevated (121.1 °C) temperatures. Clamp-up force was measured with sensor washers. In the case of room temperature tests clamp-up force decreased by an average 6% after 1000 h. In the case of elevated temperature, test relaxation was on average 10.7%. Initial clamp-up force had a minimum effect on the relaxation level. Fastener head type had no effect at all. Assuming this result, relaxation after 100,000 h was extrapolated with application of the model presented in another study [75]. It was concluded that relaxation in room temperature would achieve about 16%, whereas, in elevated temperature it could be as high as 37% for HTA matrix and 60% for KIII matrix. Therefore, long term relaxation in elevated temperature could have significant effect on bearing strength, even if short term relaxation in room temperature has small effect.

## 8. Drilling Holes

The success of the bolted joining operation depends largely on the quality of the holes. Damage-free and precise holes must be manufactured in the components to ensure high joint strength and precision [49]. Although special non-conventional machining technologies, such as electro-erosion, water-jet, and laser have been industrialised for making holes in composite laminates, conventional drilling is still the most widely used method, even though drilling of composite materials causes certain issues [76]. The hole drilling in fibre reinforced composites is highly difficult due to the characteristic features of composite materials. They are non-homogeneous, anisotropic, and contain hard and highly abrasive reinforcing fibres, as well as heat sensitive matrix [77,78,79]. Hard and abrasive fibres cause the excessive tool wear and frictional heat, whereas soft and sticky matrix clogs the tool edge, making it dull [80]. Therefore, the tool wear mechanisms during drilling composite materials are characterized by some unique features, totally different than in the case of drilling isotropic materials [49]. It is recommended to use diamond-coated or uncoated tools made of cemented carbide as well as polycrystalline diamond drills, which are more resistant to wear than steel drills when drilling composite materials [79,81]. The tool wear mechanisms during drilling composite laminates [49,79,81] are the following:

- abrasive wear,
- chipping,
- cutting edge rounding,
- micro-cracks,
- erosion,
- smearing,
- dislodgment of diamond grain,
- matrix adhesion.

**Figure 6:**
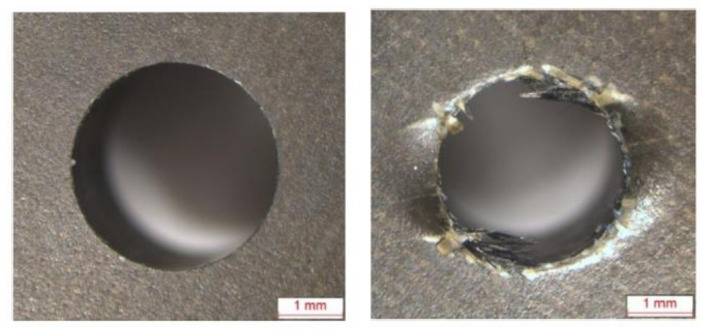
Comparison of the exit surface between holes made by a new (left) and a worn (right) tool with the same cutting parameters [89].

A lot of research works identified abrasive wear as the dominant tool wear mechanism in both conventional drilling and high speed drilling of composite laminates due to the highly abrasive nature of the carbon and glass fibres [82,83,84,85]. As reported by several researchers, tool wear leads in turn to the stronger composite damage during drilling [80,86,87,88,89]. Fernandez-Perez et al. studied the influence of the tool wear on the quality of the holes drilled in CFRP composite [89]. Carbide countersunk drill bits with diamond coating were tested. The first delamination appeared after drilling the distance of 1400 mm. In the reference cutting condition it was found that the end of the tool life, defined as the moment in which the delamination damage starts to be unacceptable, appears after drilling the total distance of 4400 mm. Figure 6 shows the differences between the holes made with a new tool and with the tool after drilling the total distance of 4400 mm in a carbon fibre reinforced laminate [89].

In a comparative wear study of uncoated and diamond coated carbide tools in drilling CFRP, Iliescu et al. [83] characterized the progression of tool wear by electron microscopy investigations and the measurement of axial forces. The diamond coating was found to increase the tool life by a factor of 10–12. The drilling of carbon fibre reinforced composite by diamond coated and uncoated drills was also studied by Gaugel et al. It was proven that, considering delamination damage as the limiting factor, the benefit of the diamond coating is a significant increase in lifetime of the drill, at least by the factor of 8 [79].

The harsh drilling process may also cause occurrence of some unexpected damages in laminate [79,90,91]:

- delamination,
- fibre pull-out,
- matrix thermal degradation,
- burrs,
- splintering,
- micro cracks.

**Figure 7:**
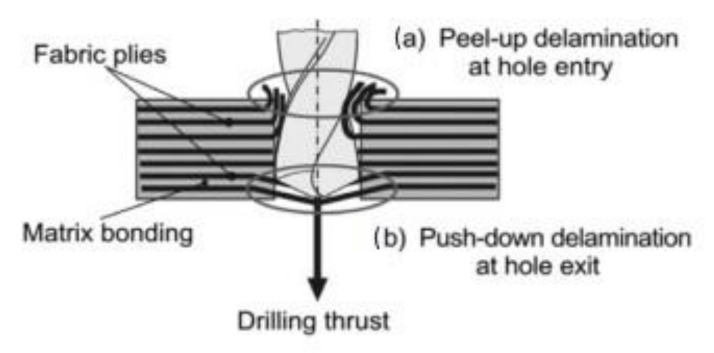
Peel-up (**a**) and push-out (**b**) delaminations [87].

In particular, delaminations, which are inter-ply failures, bring about severe and highly dangerous problems: they not only reduce drastically assembly tolerance and strength, but also have the potential for long term performance deterioration under fatigue loads [27,91,92,93]. However, there is also a research which indicates that there is no significant difference in the fatigue strength of the holed composite with visible delaminations and defect free when subjected to open-hole fatigue tensile tests [94]. The drill-induced delaminations appear both at the entry and at the exit of the holes [79,95,96,97,98,99,100,101,102,103,104]. They are called respectively “peel-up” and “push-out” delaminations (Figure 7). The peel-up delamination occurs when the drill cutting edges make contact with the composite and the peeling force directed through the slope of the drill bit flutes causes the separation of the composite plies. The push-out delamination appears when the drill bit approaches the hole exit side and the uncut plies beneath the drill become more susceptive to the deformation due to decrease of the thickness [49]. Eventually, push-out delamination appears at the drilled holes exit periphery if the thrust force applied to the uncut plies exceeds the inter-ply bonding strength [49,91,93]. Then, the interlaminar crack occurs when the interlaminar bonding strength can no longer withstand the bending deformation [91]. In practice, it has been found that the delamination associated with the push-out is more severe than that associated with peel-up [1,101].

**Figure 8:**
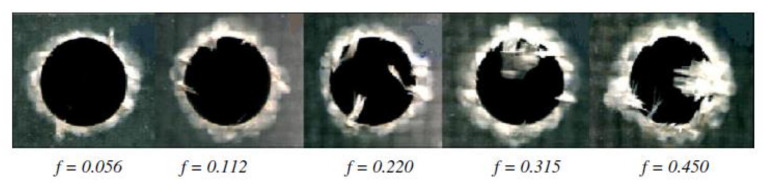
The effect of push-out delamination in GFRP composite drilled by 13-mm diameter drill for different feed rates *f* [mm/rev] and the spindle speed of 20.253 m/min [101].

The delamination size in drilled composites can be controlled by the proper selection of drilling parameters, drill geometry, and drill material [101]. Some authors proved that the feed speed and the thrust force, which depends on the feed speed [101,105], have the highest influence on the delamination magnitude [80,86,101,106,107], while cutting speed is the parameter which has the highest effect on the hole surface quality [106]. However, other authors did not observe a direct relationship between thrust force and delamination during drilling glass fibre-reinforced composite [108]. Sorrentino et al. presented a research in which they proved that for increasing both feed rate and cutting speed the peel-up and push-out delamination are exacerbated for glass- and carbon-reinforced plastic composites [102]. Khashaba et al. investigated the influence of drilling feed rate and cutting speed on the quality of the holes in woven E-glass fiber-reinforced epoxy composite laminates [101]. The specimens were machined under dry cutting conditions with five spindle speeds (6.405, 12.710, 20.253, 32.03, 50.635 m/min) and five feed rates (0.056, 0.112, 0.22, 0.315, 0.45 mm/rev) using cemented carbide drills [101]. The results indicated that both peel-up and push-out delamination increases with increasing feed rate (Figure 8). However, no clear effect for the cutting speed on peel-up or push-out delamination sizes was observed.

**Figure 9:**
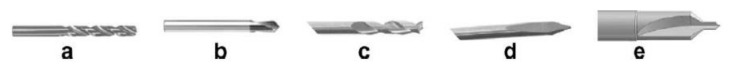
Drill bits investigated in respect of damages caused by drilling in composite laminates: (**a**) twist drill with a point angle of 120°, (**b**) twist drill with a point angle of 85°, (**c**) brad drill, (**d**) dagger drill, and (**e**) special step drill [95].

The bearing test results also show that bearing strength of the bolted joint decreases with increasing feed rate [101]. This is due to the fact that higher feed rate increases the resulting delamination damage and subsequently lowers the bearing strength. Drilling at high cutting speeds (*V* = 20.25 and 32.03 m/min) also results in lower strength compared with that machined at low cutting speed (*V* = 6.405 and 12.71 m/min) [101]. This result was attributed to the temperature increase induced at high cutting speeds which damaged the matrix [98]. The results above indicate that lower feed rate and cutting speed of drilling assure the better hole quality. However, decreasing those parameters means that the machining time would be longer, which has a significant economic impact, taking into account the huge number of holes required for the assembly of aeronautical components. Therefore, the problem of push-out delaminations was addressed by many methods. One of them was based on the change of the feed rate during dry drilling. This method consisted in reducing the feed rate as the drilling tool approaches the bottom surface of the laminate in order to decrease the thrust force and consequently the push-out delamination [102]. The push-out delamination factor was reduced in such way to 26% for GFRP and to 37% in the case of in CFRP [102]. In order to suppress the push-out delamination, the last ply is also sometimes composed of glass fibre, but this method is not very effective, especially if the cutting conditions are severe [109]. Another method widely used in the industry to reduce drilling induced delamination consists in positioning a support plate under the composite laminate in order to prevent defects due to push-out delamination [91]. This support is capable of generating a backup force that compensates the effect of the thrust force in the final step of drilling [102]. The backup force helps to suppress the delamination crack as the drill approaches the last ply, hence higher drilling thrust is needed to trigger the propagation of the delamination [91]. Tsao et al. developed an active backup support [91]. A tubular solenoid electromagnet was mounted to the composite elements subjected to drilling in order to deliver adjustable suppressing load. Once the workpiece is fixed on top of this device, the magnetic-driven backup is activated when the drilling starts [91]. The applied backup force contributes to the suppression of the growth of the delamination in carbon/epoxy laminate at drilling exit by 60–80% [91]. However, this method can only be used in the case of the elements in which accessibility is good enough to apply the support. In the structures such as pipes, the use of mechanical support is unfeasible. Hocheng et al. developed an innovative method which allows to apply the support in the case of such geometries [105]. In this method, in order to perform the drilling of a tube, magnetic colloid was mixed with iron powder and polymer colloid and applied inside the tube. An external electromagnet was placed on top of drill chuck and electrical current was passed through it [105]. The electromagnet attracts the magnetic colloid inside and introduces the back-up force from inside upward against the downward bending deformation of the last laminate ply when the drilling from outside of the tube is performed [105]. Due to the use of the new method, the delamination extent in tubular structures made of carbon fibre-reinforced polymer prepreg can be reduced by 60–80% [105]. A pre-drilled pilot hole with smaller diameter than the final hole can also be adopted in order to decrease the thrust force of the final drilling and thus the threat of delamination [110,111,112]. This method is especially useful when drilling large holes [110]. Tsao proved the influence of pilot hole on the delamination reduction during drilling holes in composite with the use of core drills [111] and saw drills [110]. Tsao and Hocheng also studied the effect of the pilot hole on the drill-induced delamination in CFRP laminate. According to these authors, the pilot hole diameter should be around 15 to 20% of the final drill diameter to minimize delamination risk [97]. In order to perform the drilling of the pilot and final hole in one operation a special step drill was designed [95]. The drill has two diameters: the smaller to drill first pilot hole and then larger to drill the hole of final diameter (Figure 9e).

The drilling-induced damages may be diminished by the correct choice of the drill geometry and material. Bonnet et al. found out that the delamination in carbon/epoxy samples can be avoided if the top angle of the drill bit is less than 110° [109]. Otherwise, delamination increases with the feed rate [109]. Jia et al. proposed a novel intermittent-sawtooth drill [113]. The theoretical and geometrical analyses of the drilling process revealed that the cutting lips of the drill could reverse the cutting direction from downward to upward and thereby, reduce the drill exit damages [113]. The tests have shown that the number of burr-free holes drilled in carbon fibre reinforced composite by the modified drill bit is nearly 6 times higher than in the case of the ordinary one-shot drill and delaminations are also suppressed [113]. Durão et al. carried out a comparative study of different drill point geometries used for carbon/epoxy laminates drilling [95]. A total of five tungsten carbide drills with 6-mm diameter and different geometries were used (Figure 9) [95]:

- a twist drill with a point angle of 120°,
- a twist drill with a point angle of 85°,
- a brad drill,
- a dagger drill,
- a special step drill.

It was concluded that thrust force varies with drill geometry and with feed rate, which enables the use of higher feed rates if adequate drill geometry is selected [95]. Lower delamination was noticed when twist drill with 120° point angle was used as well as for step drill. Taking the twist 120° drill as a basis, delamination increases by 6% for the dagger drill, 12% when using the twist 85° drill, and 14% for the brad drill [95]. Tsao and Hocheng used twist, saw, and candle-stick drills to drill carbon/epoxy composite in order to measure the damages caused by the process [104]. The saw drill and candle stick drill both punches through the last plies over a smaller area than the twist drill, thus a smaller section of the last ply is subjected to a bending force from the centre [104]. Among those three drill bits, the delamination caused by the twist drill at the same feed rate is the highest, moderate delamination is caused by the saw drill, while for the candle stick drill is the lowest. Velayudham and Krishnamurthy used three types of drill point geometries:

- conventional (connical),
- web thinned,
- tripod

to drill glass fibre reinforced composite [114]. It was found that the tripod drill performs better compared to the other drills and this geometry was found to be producing controlled thrust and torque [114]. Kumar et al. [115] used three drills with different geometries and made of different materials for drilling GFRP, namely:

- a helical flute made of high speed steel (HSS) drill,
- a carbon tipped straight shank drill made of K20 stainless steel,
- a solid carbide eight-facet drill.

The best hole quality was achieved in GFRP composite for the solid carbide eight-facet drill and the worst for the helical flute HSS drill. The carbide tipped straight shank drill made of K20 steel has the ability to dissipate heat rapidly, but the delaminations caused by it are bigger than that caused by the solid carbide eight-facet drill [115]. Durão et al. compared delaminations and bearing strengths of holes drilled in glass/carbon-epoxy laminate with the use of 5 types of drills [93]:

- HSS twist drill,
- carbide twist drill,
- carbide brad drill,
- carbide dagger drill,
- carbide step drill.

**Figure 10:**
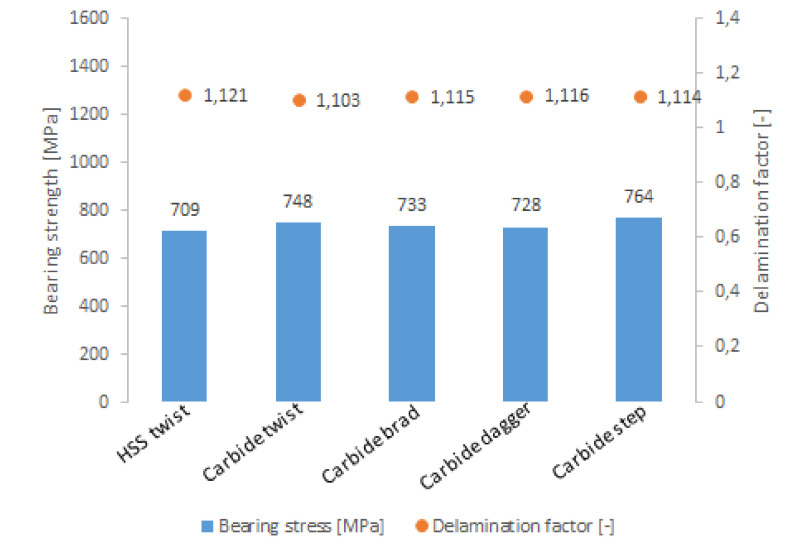
Results of drilling holes with the use of different types of drills on bearing strength and delamination [93].

The worst results of both delamination and strength gave drilling with the use of HSS twist drill. The smallest delamination was achieved with the use of the carbide twist drill; however, slightly higher bearing strength than for the carbide twist drill was achieved for the holes drilled by the carbide step drill (Figure 10) [93]. The conclusions drawn from the abovementioned works are inconsistent, which suggests that the influence of the drill shape and material on the drilling-induced damages is still a topic which demands investigation.

Another way to decrease the drilling-induced delamination may be the use of ultrasonic oscillation assisted drilling. Guputa et al. presented a research aiming to evaluate the effect of such drilling on the delamination in quasi-isotropic carbon fibre reinforced composite material with bismaleimide matrix [116]. The ultrasonic oscillations are superimposed on drill in the axial feed direction during drilling. It was found out that the delamination reduction gained from the ultrasonic assisted drilling can be as high as 25% [116].

Another problem which arises during drilling of composite materials is the heat generated by the drilling. The thermo-mechanical conditions during the drilling process are severe due to very poor thermal conductivity of the composite materials compared to metals [80]. It can make the workpiece achieve the limit temperature, beyond which the thermal degradation of the composite matrix can be triggered [117]. Very high temperatures can damage the matrix and, consequently, the laminate strength is reduced [98,118], even when overheating persists over a very short time [76,119]. It was demonstrated that if the material is exposed to the temperature beyond the cure temperature, its interlaminar shear strength is reduced [76]. For a typical epoxy-based matrix, the critical degradation temperatures range from 180 °C to 270 °C [120]. Sorrentino et al. presented a work in which the temperature near the machined surface and on the tool during dry drilling of FRP laminates was monitored [117]. Two kinds of FRP laminates were tested: one reinforced with carbon fibre and the other with glass fibre. The temperature was monitored by thermocouples placed inside the drill and the laminate. The temperature found for GFRP drilling were generally more elevated than those obtained in CFRP, because thermal conductivity of the latter is higher and so it dissipates better the generated heat [117]. For some parameters sets, the highest temperature registered in the drill was as high as 250 °C and the highest temperature in the laminate was 170 °C [117]. Both those temperatures can damage some polymer matrices, however, since the thermocouples in the laminate were placed at a certain distance from the drilled hole (1 or 3 mm), the temperature on the drill is more reliable as a measure of the temperature to which the laminate is exposed during drilling. The above findings show that the drilling process in composite can easily cause thermal damage, which may decrease the strength of the structure and is very difficult to detect. The damages can be avoided only if low drilling parameters: feed speed and cutting speed are employed. However, the use of low drilling parameters increases the time and costs of the process.

## 9. Fastener Types

Many materials can be used to manufacture fasteners. However, the most obvious ones, such as aluminum alloys and steel cannot be used to join carbon fibre reinforced composites without protecting coating or shielding, because of galvanic corrosion arising due to the difference in the potential conductivity of the composite and the aforementioned metal alloys [16,121]. A solution to avoid galvanic corrosion is to use fasteners or shields made of a metal with less difference in the galvanic potential compared to carbon-reinforced composite. Titanium alloys are a good example of such materials [122], but the fasteners made of them are expensive and heavy compared to fasteners made of aluminum alloys [16,121]. Since thousands of fasteners are usually used to join the aircraft structure, it results also in a severe cost and weight penalty [123]. However, despite these drawbacks, the titanium alloy fasteners are used most widely. To eliminate both overweight and corrosion problems, fasteners made of composite materials have been proposed. The advantages of such fasteners are the elimination of the galvanic corrosion problem, radar invisibility, low weight, and low price. These fasteners [16,121,124] are manufactured from

- carbon fibre/polyimide,
- carbon fibre/PEEK,
- carbon fibre/carbon composites.

Starikov and Schon conducted two researches in which strength of composite joints with composite bolts (PEEK/carbon) and several types of titanium bolts was evaluated in static [16] and fatigue [121] tests. In the static tests the composite bolted joint failed as the bolts sheared, whereas, the titanium bolt joint failed in the net-section failure mode [16]. The strength of the composite bolted joint was only slightly lower than the titanium bolted joints, however, as the latter failed through the net-section failure of the specimen, the strength of the titanium bolts was not fully tested. The same types of bolted joints were tested in cyclic loading to obtain the results of the joint resistance to fatigue [121]. All types of joint failed as the result of bolt fracture and the composite fasteners displayed the lowest fatigue durability. A possible explanation for this could be found in the inability of composite bolts to support shear loading [121]. Similar results were obtained by Whitworth who tested composite mechanical bolted joints with titanium and composite fasteners for fatigue [125]. Again, the composite bolts showed lower fatigue durability than their titanium counterparts. Thus, it seems that despite their advantages, composite fasteners are not likely to be used widely due to their poor strength and durability.

Another question is the shape of the bolt head. Two main types of fasteners are used in aerospace structures: with protruding head and with countersunk head. The latter type is usually used in the case of outer surface joining in the areas when the aerodynamic smoothness is of great importance [46]. The countersunk bolted joining is more costly and it weakens the structure, because of partially larger holes that have to be made to fit the bolt head inside the material. Additionally, the countersunk bolts cause asymmetrical load transfer through the bolted plates, which may lead to higher strain levels [121]. This agrees with the results from another research, where the introduction of a countersunk hole has been found to increase the stress concentration factor, particularly at the onset of the countersunk region [126]. Increasing the ratio of the countersink depth to the laminate thickness reduces the extent of bearing and promotes bending, with a change to net section failure at large ratios [15]. McCarthy et al. conducted tests on the bearing strength of carbon/epoxy bolted joint specimens with protruding and countersunk bolts [28]. For the same test conditions the bearing strength for the countersunk bolts was by 35–45% lower than for the bolts with protruding heads.

Although a circular cross-section of a fastener is an obvious choice from the manufacturing point of view, there are a few theoretical works which indicate the possibility that the non-circular cross-section would be a better solution in terms of the joint strength. Wang et al. [127] and Persson and Madenci evaluated feasibility of changing the bolt cross-section shape from circular to elliptical in order to improve the joint strength in composite materials [128]. Wang et al. performed an analysis which indicated that the joint with a bearing failure mode gained 35% strength improvement, while the joint with a shearing failure mode gained 12.9% strength improvement [127]. Persson and Madenci conducted an experimental and analytical study to compare the failure mode and stress distribution around circular and elliptical pin-loaded holes in composite laminates and also reported an increase of the bearing strength for the elliptical holes [128]. Zhou and Fei found out on the basis on the open-hole tension (OHT) specimens that the racetrack-like hole outperforms the circular and elliptical ones [129]. Zhou et al. conducted also numerical analyses in order to optimize the shape of the bolt and the bolt hole [130]. A racetrack-like shape was again chosen as an optimal shape. The net-tension and bearing stress distributions of the racetrack hole were evaluated and compared to the circular one. The comparison showed the outperformance of the racetrack-like sectioned pin: the net tension stress was lower by 28% and the bearing stress by 17% [130]. Thus, it seems that the bolts with the racetrack-like cross-section are optimal from the strength point of view. However, no feasibility studies of using such bolts in real structures have been performed yet. It seems that manufacturing such bolts and cutting non-circular holes, especially in composite, impose a significant engineering issue.

## 10. Non-Destructive Testing

**Figure 11:**
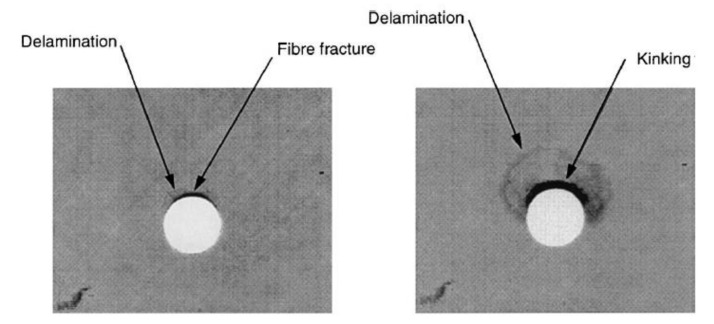
X-ray images of carbon/epoxy bolted joints after loading to different levels and disassembling [133].

The monitoring of bolted joints in composites is of great importance, because they constitute weak spots in such structures and the failure of the whole structure is likely to initiate from the bolted joint. However, techniques used most commonly for non-destructive testing of fibre reinforced polymer composites, such as:

- ultrasonic C-scans—according to ASTM E2580: Standard practice for ultrasonic testing of flat panel composites and sandwich core materials used in aerospace applications,
- shearography—according to ASTM E2581: Standard practice for shearography of polymer matrix composites and sandwich core materials in aerospace applications,
- thermography—according to ASTM E2582: Standard practice for infrared flash thermography of composite panels and repair patches used in aerospace applications,
- acoustic emission—according to ASTM E2661: Standard practice for acoustic emission examination of plate-like and flat panel composite structures used in aerospace applications,
- radiography—according to ASTM E2662: Standard practice for radiographic examination of flat panel composites and sandwich core materials used in aerospace applications

are dedicated for flat panels and are difficult to perform in the case of bolted joints due to the inaccessibility of the joint [131]. These techniques are not capable of real-time monitoring of damage evolution either [132]. However, some non-destructive techniques of monitoring were employed to assess damage development in FRP composites. Ireman et al. used microscopic examination and X-ray for inspection of bolted joined single lap shear specimens made of carbon/epoxy composite and aluminum alloy substrates [133]. Both those techniques required disassembling the joints. Microscopic examination allowed to identify fractures developing during the loading of the specimens. The hole edge was examined using a mirror angled at 45° on a bolt placed inside the bolt hole in order to check for matrix cracks in all plies, identify delaminations and to monitor their growth. A penetrant was applied to the top surface layer to bring the any fibre fracture and matrix cracking out [133]. The microscopic examination was supplied by an X-ray inspection which was also employed to reveal the damages after loading [133]. A zinc-oxide based fluid penetrant was applied to the edge of the hole, in order to reveal cracks and delaminations around the hole edge. The penetrant soaked into the cracks and thus damaged regions appeared dark on the X-ray images (Figure 11). This was especially important for assessing the area of delamination, as the microscope showed only the cracks of the delamination on the inner surface of the bolt hole and gave no indication of its depth [133].

More efficient approach than non-destructive testing is integrated approach to in-situ detection of damages called health monitoring. Several methods applicable for the health monitoring of the bolted joints in FRP composites can be found in literature. Ireman et al. used strain gauges to determine the strain distribution in the vicinity of the holes and to detect bending of the joint [133]. Nine strain gauges were placed along the radial direction of the hole on the surface acing the interface between the composite and the aluminum [133]. The changes in strain distribution around the hole allowed to determine the onset and propagation of the damage as the specimens were loaded. In the same work acoustic emission was used to detect damages during the tests of the specimens [133]. Acoustic emission sensors are used to detect cracking by sensing the energy released during crack formation [134]. One sensor of 17 mm in diameter was placed on each side of the bolt. The graphs showing number, amplitude, and duration of events as well as the load level at which they occurred were obtained [133]. This technique easily detects even micro-scale damages [134]. Yang et al. used piezoelectric transducers to investigate composite bolted joints under tensile load [135]. Such transducers are very light, thin, and can be used as actuator and sensor, respectively [136]. Guided waves generated by them are sensitive to small-scale damages and could propagate over long distance [137]. In this work Lamb wave, which is one of the guided wave modalities, was used to inspect bolted joints in fabric glass/epoxy composite. Single lap shear specimens during tensile test were investigated. Damages in the joints occurring during the tests affected the amplitude of the Lamb wave which allowed in-situ health monitoring with possibility of distinguishing failure modes. Moreover, the amplitude of the Lamb wave varied with the applied torque, which suggests that this method can be useful also for detecting of axial force loss in the bolted joints [135]. Haynes et al. investigated bearing damage in composite bolted joints using ultrasonic guided Lamb waves [138]. Circular arrays of micro-fibre sensors-actuators were bonded to quasi-isotropic CFRP composite plates. The plates were then subjected to tensile bearing tests. Optimal actuation frequency of 300 kHz and 180° interrogation angle were chosen by a parametric study. The results have shown that the method is capable of detecting bearing damage in composite as well as monitoring of the applied load if the elastic load is not exceeded [138].

Nichols et al. employed vibration-based structural health monitoring to detect damages to bolted joints in composite [139]. The structure under consideration was a beam made of glass fibre reinforced composite. Damage was introduced into the structure by reducing the preloads on both bolts connecting one end of the composite to a steel structure. Several detectors using dynamic strain response were employed as the structure was driven using simulated wave forcing. The sensing element was a Fibre Bragg Grating oriented to measure the bending strain at the beam centre approximately 10 cm from the bolts. In an effort to simulate in situ monitoring conditions, the experiments were carried out in the presence of strongly varying temperatures. The results of this work indicate that using the estimated auto-bicoherence of the systems response produced the best overall detection performance when compared to features based on a nonlinear prediction scheme and another based on information theory. For roughly 10% false alarms, the bicoherence detector gives nearly 90% probability of detection [139]. Caccese et al. presented a work in which detection of bolt load loss by structural vibration analysis was performed on a model consisting of a glass fibre reinforced vinyl ester composite panel bolted to a steel frame [140]. A piezoelectric actuator was bonded to the composite panel to provide controlled vibration input and the response of the plate was measured using either shear accelerometers or dynamic strain sensors located at the corners of the composite panel. Three different monitoring techniques were used to detect bolt load loss, including low frequency modal analysis, high frequency transfer functions between the actuator and sensors and high frequency transmittance functions between pairs of sensors. Experiments have shown that the transmittance function is the most promising approach as it was able to detect reliably a single bolt loosening. Various input frequencies from 0 to 20 kHz were analysed and it was found out that the range of 13 to 17 kHz was most sensitive. Authors claim that the damage index based on change in transmittance function is very sensitive to changes in bolt load [140].

**Figure 12:**
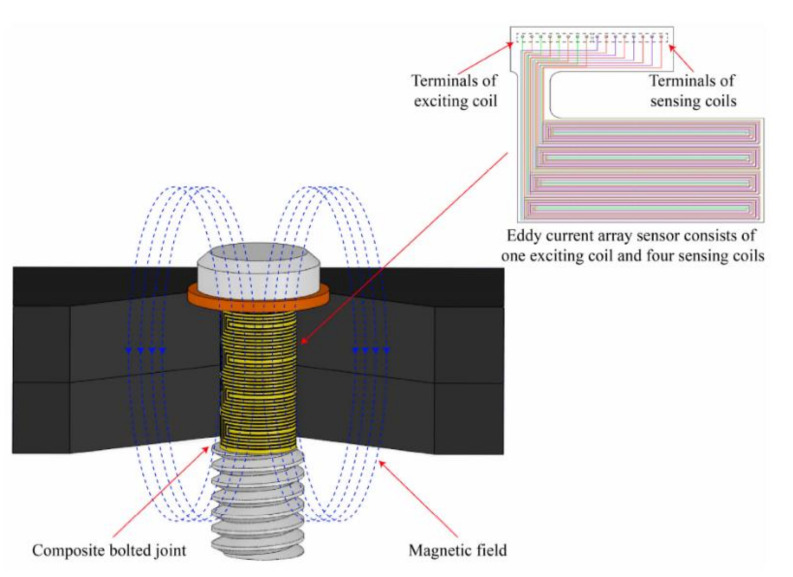
Operating principle of eddy current sensing method [131].

Due to the electrical properties of CFRP, eddy current (EC) method is practical and easy to implement [141]. Liu et al. developed eddy current array sensing film and used it for monitoring the damage around the hole-edge of composite bolted joint [131,141]. A rectangular flexible eddy current array sensing film printed on a flexible substrate was bonded around the bolt. The whole structure of eddy current senor consists of an exciting coil and a receiver coil. The principle of measurement is shown in Figure 12 [131]. The EC sensor is manufactured on a flexible substrate with printed flexible circuit technology and bonded on the bolt in composite joint. To monitor bolt hole-edge damages in both radial and axial directions, the EC sensing film is made of an exciting coil and several sensing coils [131]. According to Faraday’s Law, when an alternating electric current is applied to the exciting coil, a primary alternating magnetic field is produced and then an EC is be generated in the conductive composite material [131]. The EC generates a secondary alternating magnetic field. The occurrence of damages disturbs the generated EC due to the change of flow paths and consequently affects the secondary magnetic field. Thus, the damages in the composite can be detected [131].

The experiments on unidirectional CFRP specimens made of carbon fiber T300 prepreg were conducted. In order to make artificial defects accurately, the joint was designed to consist of four laminate plates, each 5-mm thick. The stacking sequence of each laminate plate was [0/45/90/-45]_s3_. The variation of induced voltages related to the cracks propagating along the axial direction and radial direction were investigated [131]. The results of the researches indicate that the flexible eddy current array sensor developed in this paper can inspect the damage at the hole-edge. The crack growth in both of the axial and radial directions is represented with variation value of the induced voltage [131,141]. The results demonstrated that the developed EC array sensing film can effectively identify not only whether there is damage at the hole edge, but also the damage location in the thickness and quantitative size [131]. Shimamura et al. also used in their work electric conductivity of carbon fibre reinforced composites to detect damages of bolted joints by measuring the change in electric resistance [142]. The electrodes were placed on the composite to detect bearing damage of the bolted joint during its loading. The results have shown that bearing failure of less than 10 mm^2^ causes the electric resistance change of a few hundred ppm and can be easily detected [142]. The advantages of this approach is that no additional sensor have to be embedded in composite structure, which reduces the costs of the method and that detecting electrodes can be placed comparatively far (10 mm) from the bolt [142]. On the other hand, this method can be used only in carbon fibre reinforced composites.

Thostenson and Chou and Friedrich et al. employed unique capability of carbon nanotube networks as in situ sensors for sensing local composite damage and bolt loosening in mechanically fastened glass/epoxy composite joints [132,134]. Due to the small size of carbon nanotubes, with diameters on the scale of a few nanometers, relative to the micron-sized diameters of traditional high-performance fibers, it is possible for carbon nanotubes to penetrate the matrix-rich regions surrounding fibers and create an electrically percolating network [132]. When the fibers in the composite are non-conductive, such as glass or advanced polymeric fibers, resistance changes are directly related to the accumulation of damage in the polymer matrix [132]. Specimens in single and double-lap joint configurations made of UD or cross-ply glass reinforced composite with nanotubes were tested in tension under monotonic and cyclic loading conditions [132,134]. During the testing, electrodes were attached to the specimens near the bolt and the measurements were taken by applying a constant source voltage and measuring the resulting current [132]. With initial loading, there is a linear change in electrical resistance. At approximately 60% of the ultimate load, the resistance response begins to deviate from the linear response and more noise in the measurement is noted. This constitutes a promising result as it is likely to correspond to the initial stages of bearing damage in the composite and the subsequent formation of longitudinal cracks. At peak load there is a sharp knee in the resistance response followed by a further increases in resistance as the material is sheared-out [132]. In the case of cross-ply composite, joints experience multiple damage mechanisms and the method employing carbon nanotube networks seems to be capable not only to detect the onset and propagation of failure, but even indicate the failure mechanism [134].

## 11. Techniques for the Improvement of Bolted Joint Performance

Due to many problems arising in composite bolted joining, which deteriorate the performance of the joints, some ways to improve their efficiency have been developed. Those ways are described in the following sections.

### 11.1. Metal Bonded Inserts

**Figure 13:**
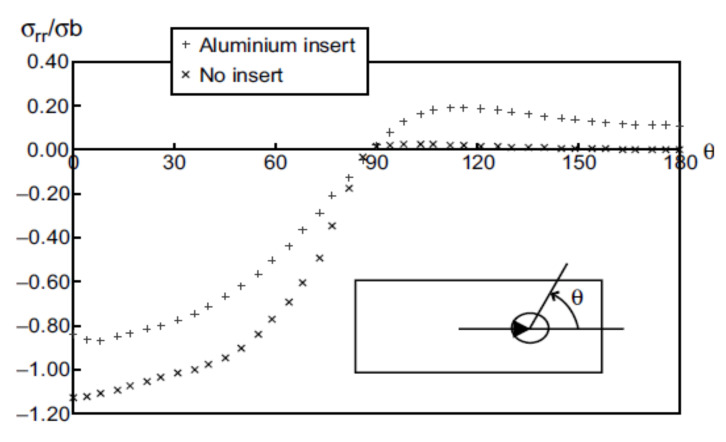
Distribution of normalised radial stresses against the angular position around the hole in the laminate [18].

The use of metallic inserts bonded to the laminate around the hole is a technique designed to increase the efficiency of composite bolted joints [18]. Metallic bonded inserts modify the way in which the load is transferred to the laminate: the radial tractions due to the bolt-hole contact are transferred using the whole surface of the composite hole instead of approximately half of the hole surface [18]. The load transfer from the bolt into the laminate for double-shear joints with and without inserts are presented in Figure 13, where the normalized radial stress is plotted as a function of the angular position around the hole of the laminate. The radial stress, *σ*_rr_, is normalized using the bearing stress *σ*^b^, defined as the ratio of maximum bearing force to the cross-section area of the bolt hole [18].

**Figure 14:**
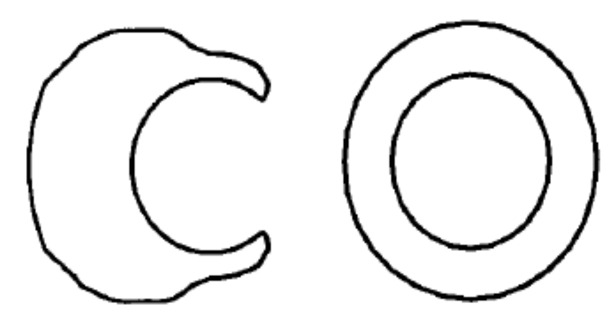
FEM-optimized and circular shape of metal insert [144].

It can be seen that the lowest radial stresses occur in the vicinity of θ = 0°. When the bonded inserts are used the stress is redistributed partially on the edge of the hole for 90° < θ < 180°, where without the insert the radial stress would be close to 0 [18]. Based on those findings, it is expected that composite bolted joints with bonded metallic inserts have higher bearing strengths than the joints without any inserts. However, some experimental results showed no improvement of the joint strength when bonded inserts are used [143]. The reason for the absence of the improvement was explained by the simulation of the behaviour of a bolted joint with bonded inserts in carbon-fibre reinforced plastic. It was concluded that the adhesive which bonds the insert fails before the full strength of the joint is obtained [143]. It was also shown that the aluminum inserts are preferable to the steel ones, because besides the lower weight penalty they cause lower stresses in the adhesive [143]. Rispler et al. proposed the bonding of a metallic insert in the highly stressed bearing region in a bolted joint in composite material [144]. The insert provides a localised plastic zone, which reduces the stress concentration on the composite material providing stress relief [144]. Two kinds of aluminum alloy inserts were used: finite element method (FEM)optimized and circular (Figure 14).

**Figure 15:**
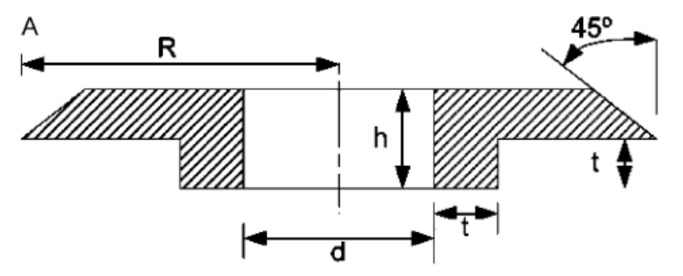
Optimized geometry of metal insert [18].

The inserts were bonded by an adhesive to the surface of composite panels around holes with diameters of 14 mm. The reduction of bearing stress concentration was verified by photoelastic bearing experiments. A 55% reduction of maximum shear strain with the optimized insert was proved when compared to the strain concentration of the specimens with circular inserts [144]. Camanho et al. also performed a numerical analysis to optimise the shape of an aluminum alloy insert used to improve the efficiency of single-shear bolted joints in composite made of carbon/epoxy laminate [18]. The optimized geometry is shown in Figure 15.

Experiments were carried out in order to validate the numerical results. In order to compare the mechanical behaviour of single-shear joints with and without bonded inserts, experimental tests were performed. Both configurations with and without inserts failed by bearing. However, in the case of the specimens with inserts, the bearing occurred after debonding between the insert and the composite. The maximum strength of the joint was increased by 24% when the inserts were used [18], which is not a very significant improvement, taking into account the significant weight penalty caused by adding the insert to the joint.

**Figure 16:**
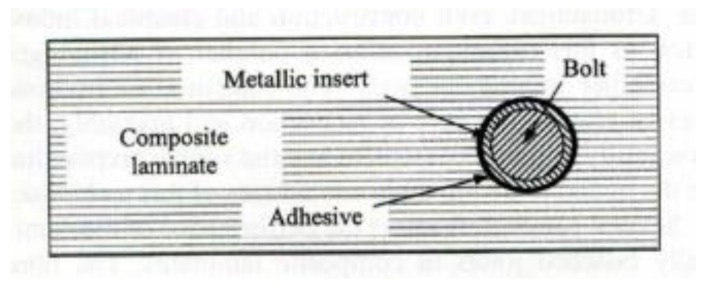
Metallic insert between the bolt and the laminate [18].

Another type of metallic inserts placed between the bolt and the laminate has been proposed and tested experimentally by several researchers. Such inserts are usually bonded to the inner surface of the hole (Figure 16).

Herrera-Franco and Cloud determined the surface strains of double shear bolted joints in cross-ply GFRP with and without bonded inserts with the use of Moiré method [145]. Aluminum alloy was used for the 1.6-mm thick inserts bonded in the 9.5-mm holes in specimens. The use of aluminium inserts allowed to decrease the maximum bearing, tensile and shear stress by 75%, 38%, and 71% respectively [145]. The experiments have shown that the adhesive failed first, but even after this the stresses obtained were still lower than those obtained for a hole without the insert [145]. Nilsson compared the strength of double shear bolted joints in CFRP with and without 2-mm thick aluminum and steel inserts bonded inside 14 mm holes [146]. The use of aluminum inserts increased the bearing strength by 12%, while the use of steel inserts reduced the strength by 6% [146]. Mirabella and Galea performed an experimental investigation on the strength of bolted joints in CFRP specimens using different aluminium inserts [147]. Insert thickness of approximately 1 mm and hole diameters of 4.2 and 6.35 mm were used. The use of the straight aluminum inserts decreased the bearing strength of the specimens by 65%, but the use of the top-hat aluminum inserts with tapered ends increased the strength by 21% [147]. The top-hat inserts are reported to increase the strength by providing new load paths and by reducing the fastener rotation [147]. However, no explanation of the premature failure of the specimens with straight aluminum inserts was provided. After performing numerical analysis Camanho and Matthews concluded that the use of thinner inserts and more compliant insert materials, e. g. aluminum instead of steel, is recommended, because stiffer and thicker inserts may create premature damage in the laminate and decrease in the joint strength [143]. This conclusion corresponds with some of the experimental findings cited above.

The above results indicate, that the strength of the joint with metal inserts could be improved if a better way of bonding metal inserts to laminates was invented. Additional advantage of the use of bonded metallic inserts inside the holes in composite, apart from redistribution of the stresses acting on the hole, is shielding the holes from damage caused by repeated installation of the fasteners [143]. Moreover, damaged holes in the composite material can be repaired by bonding such inserts [143]. The use of metallic bonded inserts inside the holes in CFRP allows also to avoid the use of titanium fasteners. When a bonded insert (such as presented in Figure 15) is used, the composite is in contact with an adhesive and not with a metallic part that may promote galvanic corrosion [18]. The disassemble of the joint with metal inserts is still possible when a separate insert is bonded to each lap of the joint [18], but the disadvantage of such a solution may be that additional insert means the increase of the structure mass. However, the bonded inserts may substitute the washers, which are often used in bolted joints [25], and thus mitigate the weight penalty.

Apart from metal inserts composite bonded inserts were also investigated. Muc and Ulatowska proposed bonding of composite insert to the bolt hole in composite material in order to decrease the stress concentration around it and thus increase its net-tension strength [148]. It is assumed that the reinforced insert is made of curved fibre composite so that the fibre orientation may vary from point to point. Finite element analysis of a composite plate containing a hole with insert was carried out. The obtained results were encouraging indicating 37% of the strength rise in the case of a plate with 0^o^ directed reinforcement and 49% of the strength rise in the case of a plate with 45° directed reinforcement. Moreover, the weight penalty in the case of composite inserts will be lower than in the case of metal inserts. However, although the design of structures with curved fibre inserts seems to be promising and efficient from theoretical point of view, it still cannot be considered as cost efficient [148], because forming of a circular insert with continuous fibres remains challenging. Moreover, the question of the composite bonded inserts definitely needs experimental investigation if it is to be utilized in real structures.

Effect of metal bonded inserts on performance of bolted joints is summarized in Table 1.

### 11.2. Titanium Foil Internal Inserts

**Figure 17:**
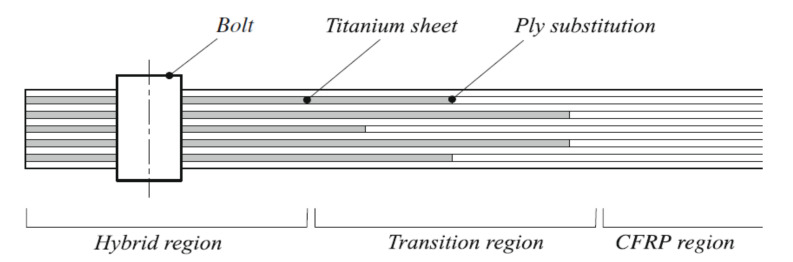
Hybrid CFRP/titanium composite at the bolted joint location [19].

A common way to improve the strength of bolted joints is local laminate build-up [19,149]. The resulting increase of laminate thickness leads to additional laminate stresses due to eccentricities, particularly in the case of single-shear joints, which are common in aerospace structures [19,20,149]. Additionally, significant weight increase due to additional composite plies, larger grip lengths, larger bolt diameters, and heavier metallic fittings is observed [19,20,149]. An alternative technique to the local build up is the substitution of some of the composite plies with titanium foil. This technique was described in several works [19,20,149,150]. The technique applied to the region in the vicinity of the bolt is accomplished by the gradual substitution of specific composite plies by titanium foils within the transition region (Figure 17). The remaining composite plies are not interrupted and pass from the pure composite region through the transition region to the hybrid region, thus acting as adhesion interlayers between each embedded metal foil [19,20].

The local implementation of metal foils, which replace composite plies, results in material with high bearing capabilities, shear, notched tension, and compression strengths in the hybrid region, which are indispensable to obtain high bolted joint effectiveness [20]. The use of hybrid composite increases particularly the bearing strength, the joint stiffness and reduces the sensitivity of the mechanical properties to the laminate configuration and environmental effects [19]. Moreover, the material takes advantage of the favourable isotropic behaviour of metal compared to the complex stress state of the bolt loaded holes in pure FRP composite [20].

**Figure 18:**
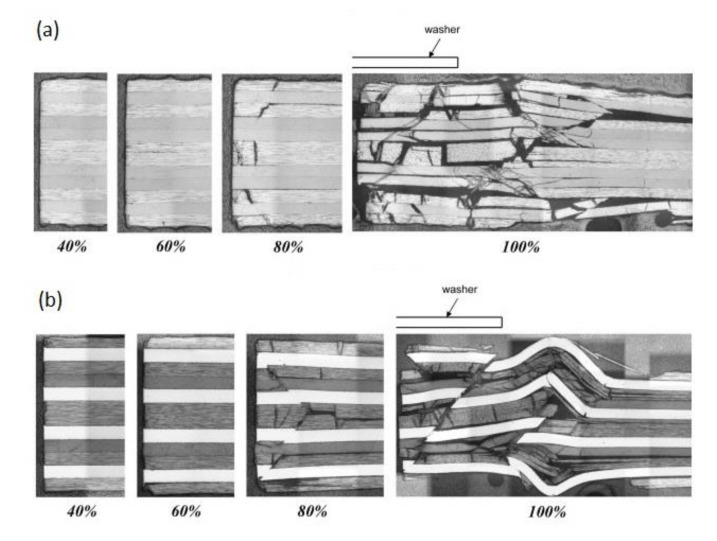
Micrographs of the bearing plane of (**a**) pure and (**b**) hybrid laminate at different percentages of the peak load [19].

Camanho et al. carried out tests for two different types of specimens, one representing the bearing region and the other representing the transition region for different contents of the titanium plies in the CFRP laminate [19]. The results show that the bearing strength increases with the titanium content. There is remarkable improvement of 158% in bearing strength for 50% titanium content in the laminate compared to the baseline specimens without titanium plies. The specific bearing strength of the joint, defined as the ratio between the bearing strength and the mass of the joint, increases by 29%. It was also observed that the joint does not fail prematurely at the transition region, which means that the measured strength of the bolted joint specimens will be the same in the case of composite structures. Fink and Kolesnikov also conducted bearing tests of carbon reinforced composite specimens with different titanium contents [149]. The static tests were conducted up to the failure in on-axis and 90°-of-axis loading directions. The test results showed that the titanium reinforced composite material offers a strength increase of about 80% under on-axis loading and 180% under 90°-of-axis loading compared to the bearing properties of the carbon reinforced laminate [149]. Hybrid material containing 54% titanium offers similar bearing strength to titanium alloy. It was also demonstrated that the use of hybrid laminates with 20% titanium content increases the tensile strength of a three-row bolted joint by 91% compared to that of a full carbon fibre reinforced plastic (CFRP) laminate, whereas the specific tensile strength is increased by 32% [149]. Camanho et al. also investigated the influence of the 16.7%, 33.3% and 50% titanium foil content in carbon fibre reinforced epoxy composite on the bolt bearing strength on-axis and 90°-off-axis [19]. The failure mechanisms are clearly visible in the through-the-thickness micrographs of the bearing plane of the hybrid specimen with 33.3% titanium content shown in Figure 18 at different load levels with respect to the ultimate bearing strength [19]. The ultimate bearing failure is triggered by the damage accumulation within the washer-supported area and the damage propagation within the unsupported area leading to extended delamination and out-of-plane deformation of the metal sheets [19].

**Figure 19:**
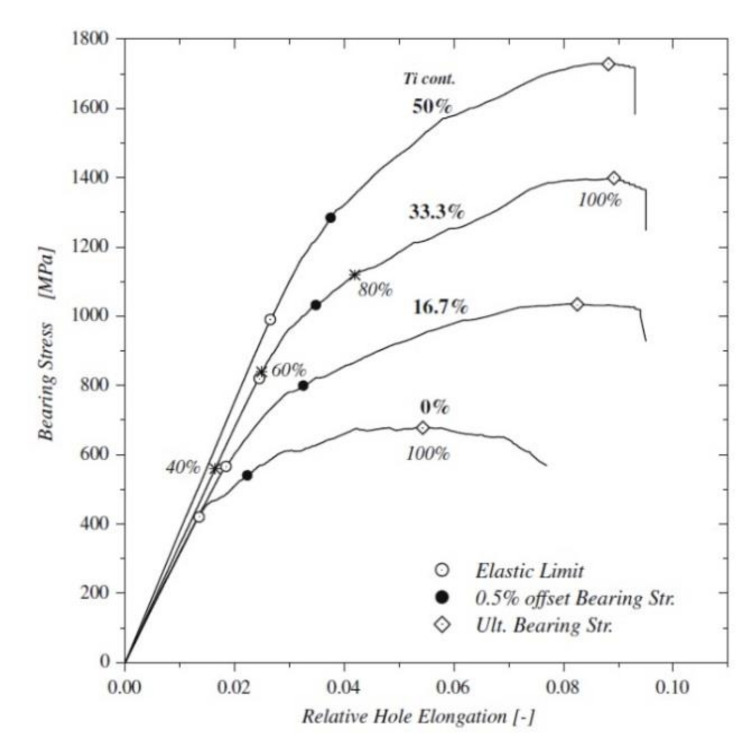
Strength curves of bearing tests for different contents of titanium foils in carbon/epoxy laminate [20].

The bearing tests of hybrid titanium/carbon/epoxy composite laminate were conducted also by Fink et al. [20]. The stress/strain test curves for 0%, 16.7%, 33,3%, and 50% titanium content are presented in Figure 19. They show clearly that the increase of titanium content to 50% causes the bearing strength increase to approx. 1700 MPa from approx. 650 MPa for pure carbon/epoxy composite [20].

The pull-out ultimate strength was also tested and the improvement by a factor of 1.3 and 1.8 for a titanium content of 16.7% and 33.3% in regard of 0% titanium content was observed [20]. Kolesnikov et al. investigated replacing some plies in UD CFRP prepreg laminate with titanium foils [150]. In order to evaluate the impact of the titanium content on the load capability of bolted joints, single-row and three-row bolted joints were tested considering different titanium contents. The maximum strength improvement compared to the reference composite bolted joint was 91% (41.5% specific strength) achieved for one bolt configuration and for 54% titanium content [150]. Fink et al. also investigated bolted joints of hybrid titanium/CFRP UD prepreg [151]. The investigated titanium content was 54.5%. The hybrid material in comparison to pure composite offered a bearing strength about 2.8 times higher than that of 70/20/10 carbon composite with 70% of 0° plies, 20% of 45° plies, and 10% of 90° plies and 4.3 times higher than that of unidirectional (0°) laminate [151].

The use of titanium as the reinforcing metal in the abovementioned works was caused mainly by its high compatibility with carbon fibres. Titanium has also good specific mechanical properties, is resistant to corrosion and has a relatively low coefficient of thermal expansion [19,149,150]. The low coefficient of thermal expansion of titanium in comparison to other metals leads to moderate residual thermal stresses induced during curing at high temperatures and cooling down to room temperature. Those stresses, however, must not be neglected, since the thermal expansion of the titanium is still significantly higher than that of carbon fibre reinforced composite. Experimental investigation in which the fracture resistance of the adhesion interface between the pre-treated titanium (surface cleaning and chemical pickling) and epoxy resin was evaluated by means of mode II fracture in-plane shear tests [149]. Short beam tests were run under the combined effect of temperature and moisture exposure. The results showed lower interlaminar shear values measured for the hybrid material. It was explained by the authors as the superposed effect of thermal residual stresses [149]. However, no direct investigation was carried out to evaluate the influence of the difference in thermal expansion coefficients of the components of hybrid composites on their strength. Another obvious disadvantages of the method is the increase of the mass, because titanium foils are heavier than the plies of the composite they replace, and the complication of the manufacturing process. Especially, drilling holes in the hybrid composite seems to be a challenging task.

Due to its high absolute strength and low material cost, stainless steel is also considered as a reinforcement material. However, its higher thermal expansion coefficient, higher density and its electrochemical sensitivity to carbon constitute some disadvantages [149] and thus no research on the use of steel foils in hybrid composites has been made so far.

The effect of titanium foil inserts on performance of bolted joints is summarized in Table 2.

### 11.3. Fibre Steering Technique

**Figure 20:**
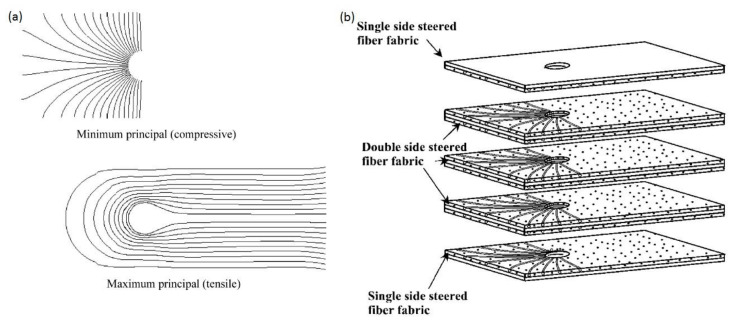
(**a**) Principal stress trajectories of a pin loaded laminate, (**b**) Typical lay-up of steered fibre samples [26].

The high strength and stiffness of carbon fibres is lost if holes or other notches are cut in the laminate disrupting the load carrying fibres [152]. The fibre steering is a technique developed to counteract this undesirable phenomenon. In this technique an improvement in stiffness and strength can be expected if fibres can be steered to match the paths by which the load traverses the structure [26,152]. However, the steered fibre bunches must be accurately lined up with the load flow direction to sustain the maximum performance [26]. The fibre steering technique was reported by several authors [26,40,152]. Continuous trajectories were defined following the principal stress vectors determined from a finite element analysis (Figure 20a) [26,40] or load paths [40,152]. Load paths are defined as regions in which the load in a selected direction remains constant from the point of application in a structure through to the point of reaction outside the structure [152]. However, there is very little difference between the tensile principal stress trajectories and the dominant load path trajectories [152]. The trajectories were digitised and input to a Cartesian robot carrying a fibre placement head or made as templates by manual placement. In the latter method, dry fibres were laid onto a tacky surface of prepreg fabric at room temperature [26,152].

According to Li et al., the peak load of bolted joint in carbon/epoxy composite coupons has been increased by 169% and bearing strength by 36% for the modified steered pattern compared to unmodified specimens [26]. However, as the steer fibre pattern (Figure 20a) was added between every two of 5 composite plies in the specimen (Figure 20b), this strength improvement was followed by a severe weight penalty. Tosh and Kelly tested two kinds of specimens made of carbon/epoxy composite with load path pattern: specimen containing an open hole and specimen with a pin-loaded hole [152]. The ultimate failure loads of each specimen were normalised for differences in weight, giving ultimate specific failure loads. Specific strength increases of 62% and 85%, respectively for open hole and pin-loaded specimens were achieved [152]. Crosky et al. used both fibre steering patterns: principal stress and load path to improve the bearing strength of carbon and glass reinforced composite specimens with holes [40]. In the load path pattern a single layer of steered fibres was used to simultaneously enhance both the tension and compression properties. The principal stress pattern produced more substantial increases of 169% (peak load) and 36% (bearing strength) [40,152]. However, no significant improvement was obtained when steered carbon fibre along principal stress patterns were incorporated into glass fibre laminates [40]. It was explained that the steered carbon fibres in this case, because of their higher modulus and lower strain to failure, failed before the bearing strength of the fibre glass laminate was achieved, thus imparting no improvement of the strength of the specimens [40]. The carbon specimens steered with the load path pattern gave a 53% increase in peak load and a 33% increase in bearing strength. In contrary to what was observed for the principal stress trajectory specimens, which failed in the net-section mode, failure in the load path specimens occurred in bearing, indicating that the load path patterns provided surplus net section strengthening [40]. Although the increase in strength of the principal stress patterned specimens was higher than in the case of the load path patterned specimens, it is possible that the results for the specific strength would be different, because in the former case the pattern requires two layers of steered fibres, whereas in the latter only one layer.

The effect of fibre steering on performance of bolted joints is summarized in Table 3.

### 11.4. Additional Reinforcement

Adding particle reinforcement to the resin is a common technique used for improving mechanical properties of polymer matrices used in fibre reinforced composites [153]. The addition of particles to the composite resin may have also positive effect on the bearing strength of the composite caused by the fact that the dispersed filler particles act as mechanical interlocking between fibres and epoxy matrix which increases friction coefficient [153]. This may prevent interphase cracking in the composite. Moreover, under a compressive loading, the fillers apparently improve the load bearing capability of a composite [153]. Asi investigated experimentally the bearing strength behaviour of pinned joints in glass fibre reinforced epoxy composite filled with different proportions of Al_2_O_3_ particles [153]. The weight fractions of the filler in the matrix were 7.5%, 10% and 15%. The increase of the Al_2_O_3_ particle content in the matrix up to 10% allowed to improve the bearing strength of the composite by 20% in regard of unfilled resin specimens [153]. Further increases in the Al_2_O_3_ particle content in the matrix resulted in slight decrease of the bearing strength. The author attributes the decrease of strength with the increasing particle content to the possibility of the appearance of large agglomerates of particles in the matrix which deteriorate its mechanical properties [153]. Crosky et al. utilized the idea, based on Sun and Jun microbuckling model [154], that the stiffening of the matrix should improve bearing performance of the bolted holes [40,155]. Initially nancomposites were prepared from the epoxy resin with clay loadings of 1–20 parts per hundred of resin (phr), cured and tested in uniaxial compression [40,155]. The compression modulus was found to increase progressively with increasing clay content. The nanocomposite reinforced with 20 nanoclay parts phr showed a 50% increase in modulus compared with the pure resin. Bidirectional 0°/90° laminates were prepared from 20 piles of carbon fibre woven fabric impregnated with neat resin and also with resin reinforced with 7.5 and 12.5 nanoclay parts phr. The laminates were then tested using the bearing test. The results of the tests have shown that no improvement in bearing strength was obtained at either clay reinforcement level. However, it was noted that the load displacement curves were steeper for the nanoclay reinforced laminates with the bearing stiffness being increased by 5% for 7.5 nanoclay parts phr and 15% for 12.5 nanoclay parts phr [40,155]. The strain to failure was lower for the nanoclay reinforced composites. Samples of the three composite types were examined using scanning electron microscopy. It was noticed during the examination that the nanoclay reinforced composites showed a much higher level of matrix failure than the composites made with unreinforced resin. This indicates that the presence of the nanoclay particles had weakened the resin and changed the failure mode, which is consistent with the reduced strain to failure observed for the nanoclay reinforced laminates [40,155]. Those results indicate that the incorporation of the nanoclay into the matrix resin introduced a premature failure mode and the advantages gained by matrix stiffening by nanoclay could be utilized only if a way to mitigate this failure mode was found.

**Figure 21:**
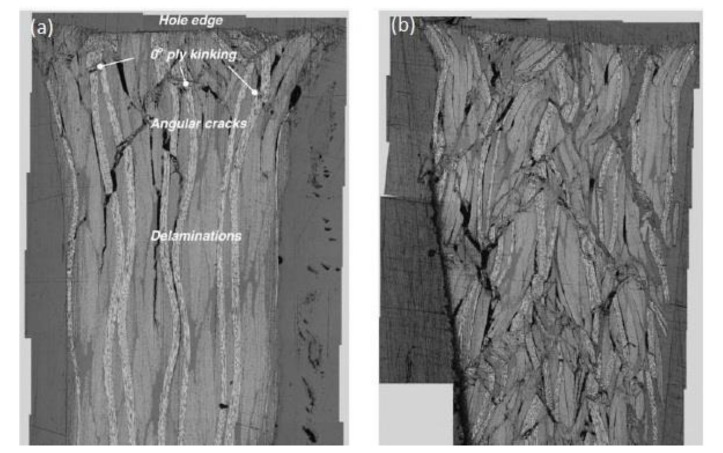
Section view of the laminate without pins: (**a**) immediately after initial bearing failure and (**b**) after loading well beyond the maximum bearing load [156].

**Figure 22:**
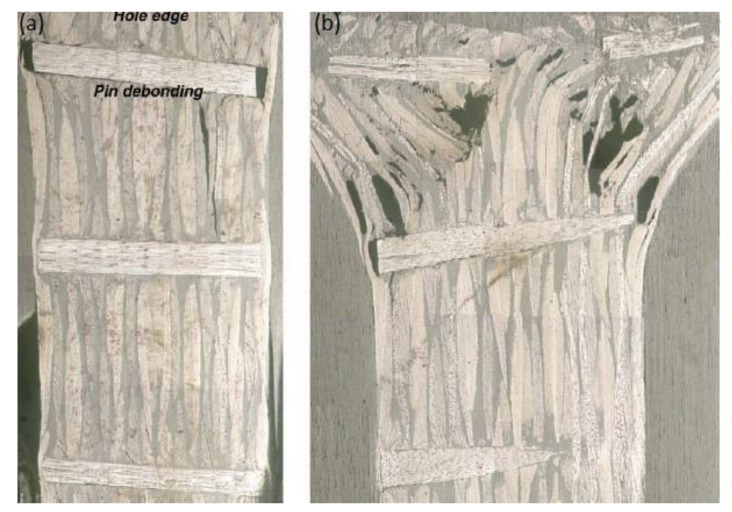
Section view of the laminate containing 2% carbon reinforced pins: (**a**) immediately after initial bearing failure and (**b**) after loading well beyond the maximum bearing load [156].

The through-the-thickness reinforcement accomplished by inserting z-pins into laminate has been designed in order to increase the delamination toughness, impact damage tolerance and ultimate strength of structural joints [156,157,158]. The z-pins are thin rods made of high strength alloy or fibrous composite embedded in the through-the-thickness direction of laminates in volume contents typically in the range of 0.5–5% [158]. The composite bolted joints are susceptible to crushing and delamination damage near bolt holes [156]. The z-pinning is expected to improve the situation by suppressing those damages around the bolt holes and thus to improve the bearing strength [156]. Through-the-thickness reinforcement made of z-pins was examined by Crosky et al. [40]. Baseline (unpinned) and z-pinned 16 ply quasi-isotropic laminates were prepared from plain weave fabric prepreg. The z-pins were made of carbon fibre and had the diameter of 0.28 mm. The use of Z-pins increased the ultimate strength by 7%, but caused the 6% reduction of bearing strength. The authors attribute this loss in bearing strength to the fact that the laminate bulked out in the region of the z-pins, compared to the rest of the laminate during curing and that the additional bulk consists of additional amount of resin which is responsible for the decrease of bearing strength [40]. Li et al. investigated the effect of z-pins on the bearing properties and damage tolerance of bolted joints in carbon/epoxy composite [156]. The region around bolt-holes in carbon/epoxy laminates was reinforced in the through-thickness direction with different volume contents and sizes of fibrous z-pins before curing. Bearing test results show that the z-pins improved the bearing stiffness (by 7.5–9.6%), ultimate load (by 7.7–12.8%), failure strength (by 7.4–9.8%), and elastic strain energy absorption (by 8.5–16.3%) of the composite joints [156]. The bearing properties increased at a quasi-linear rate with the z-pin content, but were not dependent on pin diameter. Stiffness is improved by z-pins by increasing the through-the-thickness tensile modulus around the bolt hole of the joint. Post mortem microstructural examination of the failed joint specimens revealed that z-pins improve the bearing strength by reducing cracking near the bolt hole via an interlaminar bridging mechanism that involves debonding and frictional sliding of pins within the damaged region [156]. Figure 21 and Figure 22 present the micrographs of failed specimens with and without z-pins through-the-thickness immediately after initial bearing failure and after loading well beyond the maximum bearing load [156]. It can be noticed that in the case of unreinforced laminate in the onset of failure delaminations, cracks and kinking of the plies appear and they propagate through the laminate as the load is being increased (Figure 21). In the case of z-pinned reinforced laminate, no such damages occur in the onset of failure, but the first damage is the debonding of the pin closest to the hole [156]. As the load was increased, the pin cracked and the laminate around it was severely damaged, but the next pin prevented further spreading of the damages (Figure 22).

**Figure 23:**
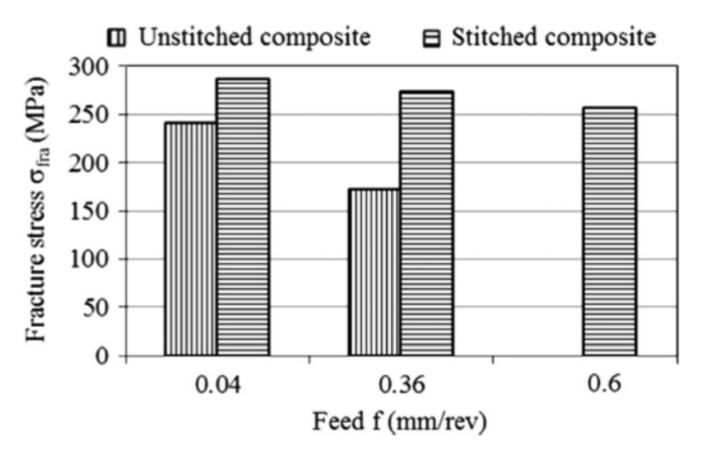
Effect of drilling feed rate on the open-hole tension (OHT) strength of unstitched and stitched composites [90].

Turki et al. used through-the-thickness stitching by a carbon thread, in order to improve open-hole strength of quasi-isotropic carbon/epoxy composite [90]. This method has been used earlier to enhance the delamination resistance of composites [159]. The composite has been stitched firstly and then the resin was transferred to it by infusion [90]. Six-mm holes have been drilled in the composite. To assess the drilling effect on mechanical properties of the composite, tensile tests of stitched and unstitched composites have been performed. Figure 23 shows the evolution of the ultimate stress as a function of drilling feed rate for unstitched and stitched specimens. It can be seen that for both stitched and unstitched specimens the ultimate strength decreases with the increasing feed rate, but the stitching improves the strength significantly [90].

**Figure 24:**
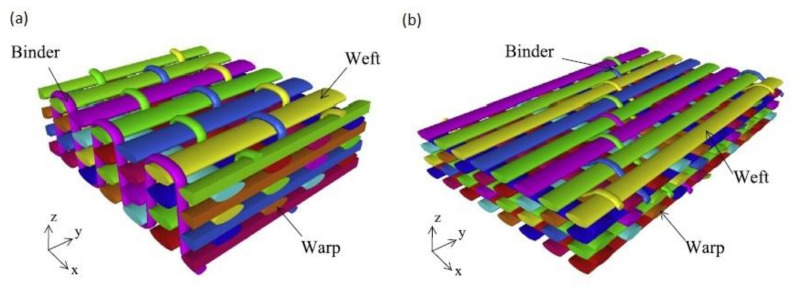
Two types of composite stitching: (**a**) through-the-thickness stitching 1-by-1 orthogonal weave W-1 and (**b**) through-the-thickness angle interlock weave W-3 (right) [160].

The stitching-induced difference in laminate failure is clearly seen in microscopic pictures. The main mode of failure changes from delamination for unstitched specimens to fibre fracture for stitched specimens. Therefore, it was observed that the stitches prevent the propagation of delaminations [90]. Dai et al. also examined the influence of the through-the-thickness stitching on the open-hole strength of carbon fibre reinforced composites [160]. A 1-by-1 orthogonal weave (W-1) and a through-the-thickness angle interlock weave (W-3) reinforcements were added to the composites. Figure 24 shows the idealised weave geometries.

Two hole diameters were used in the OHT test: 4.1 mm and 12.5 mm. The open-hole quasi-static tensile tests revealed that W-1 stitched specimens exhibited higher reduction in its tensile strength compared to un-notched specimen (17–20%) than W-3 stitched specimens (1–9%), and surprisingly using a larger hole diameter had little effect on the tensile strength (less than 10%) [160]. However, no comparison with the strength of unstitched specimens was made. Yudhanto al. investigated the tension and compression response of open-hole carbon/epoxy specimens reinforced using Kevlar-29 yarns in through-the-thickness direction [161]. The hole diameter was 5.7 mm. Two types of stitch were used: around the hole and parallel to the hole. For the round stitch configuration, the hole is surrounded by a circular stitch line of 7-mm diameter. For the parallel stitch, the distance between two stitch lines is 15 mm [161]. The static and fatigue tests were performed in tension-tension and compression-compression modes. No strength improvement for stitched specimens was observed for static tests in comparison to unstitched specimens. In the case of the fatigue tests the results were even worse: the stitching seems to deteriorate the fatigue strength in most of the cases. The exemption is compression-compression fatigue test of the parallel stitch specimens, which proved significant fatigue strength increase compared to unstitched specimens. Han et al. also investigated the influence of Kevlar yarn stitching on stress concentration of carbon/epoxy specimens with 60 mm diameter hole [162]. 1 or 2 stiches were placed around the hole and open hole tension tests were performed. It was found that the stitching decreased the stress concentration factor only slightly compared to unstitched specimens.

The effect of additional reinforcement on performance of bolted joints is summarized in Table 4.

### 11.5. Moulded Holes

**Figure 25:**
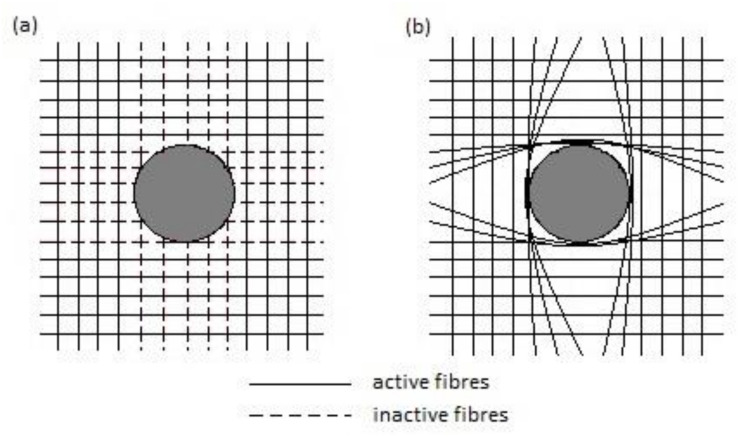
Load-bearing fibres for composites with (**a**) drilled hole and (**b**) moulded hole.

Drilling cuts the fibres coinciding with the hole, so these fibres become inactive in terms of load bearing [163]. The number of those coinciding fibres increases with the increasing hole diameter [163]. The solution of this problem is manufacturing holes by aligning reinforcing fibres around the holes before the composite is cured. Thus, the fibres remain continuous and may still take active part in the load transfer (Figure 25) [163].

As such holes are manufactured in the stage of laying plies in moulds, at least in the case of thermoset composites, they are most commonly known as “moulded” or “moulded-in” holes [164,165,166,167]. The fibre yarns at the vicinity of the moulded hole are closely packed, so while the continuity is maintained, the local fibre volume content is increased [164,165,168].

**Figure 26:**
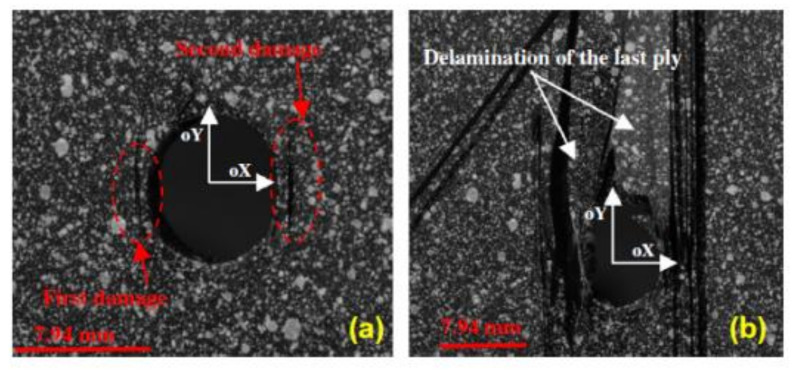
Damage progression for different loading levels for 7.94 mm moulded hole in OHT test for (**a**) 47 kN and (**b**) 64 kN loading [164].

**Figure 27:**
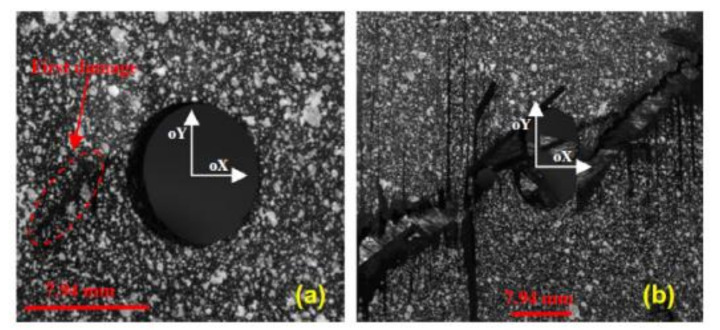
Damage progression for different loading levels for 7.94 mm drilled hole in OHT test for (**a**) 42.5 kN and (**b**) 49 kN loading [164].

Sevkat et al. compared the bearing strengths of the specimens made of plain weave E-glass fabrics manufactured by vacuum assisted resin transfer moulding (VARTM) with drilled and moulded holes [163]. A special metal mould with removable pins was designed and manufactured during this study. Dry woven-glass fabrics were stacked on the metal mould and the fibres were aligned around the pins. Prior to laminating, pins were waxed with the mould release agent so they could be removed after the solidification of the laminate [163]. Specimens with different width to hole diameter (w/d) ratio were prepared. The specimens with moulded holes were compared with their counterparts with drilled holes. The average bearing strength increase in the case of the specimens with moulded holes was 12%, 25%, 28%, and 24% for w/d ratios of 4.23, 3.39, 2.82, and 2.37, respectively [163]. However, the specimens with drilled holes for w/d ratios 2.80 and 2.37 exhibited net-tension failure, so the bearing strength increase in the case of those two types of specimens is not entirely appreciated. Zitroune et al. conducted open-hole tensile tests of the specimens made of unidirectional carbon/epoxy prepreg with moulded and drilled holes [164]. The test results showed that the strength of the moulded hole specimens is higher than those obtained for drilled hole specimens [164]. This difference is higher or equal to 30%. The specimens with the moulded holes showed totally different fracture mechanism than those with the drilled holes (Figure 26 and Figure 27) [164]. For the specimens with drilled holes a sudden fracture across the fibres was noticed and for the specimens with moulded holes a progressive fracture along the fibres was observed. The authors attribute the change of the failure mode to the increase of fibre content in the vicinity of the moulded holes.

**Figure 28:**
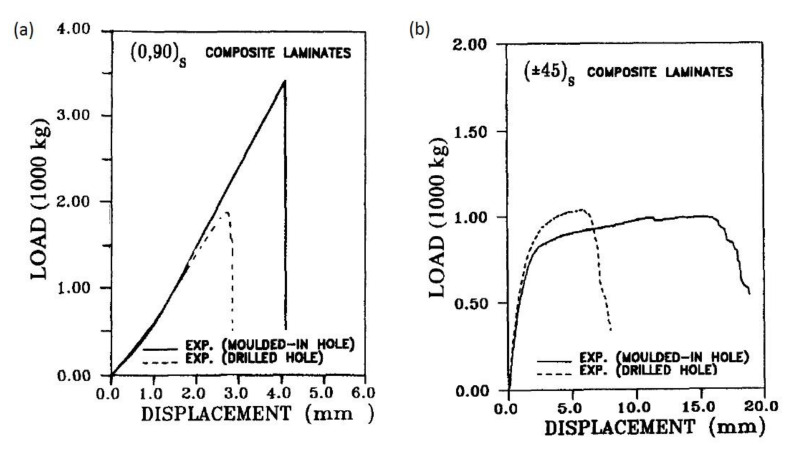
Typical load/displacement relationships for OHT tests with moulded and drilled holes for (**a**) [0,90]_S_ laminate and (**b**) laminate [±45]_S_ [166].

It seems that it is advantageous to employ moulded holes in woven fabric composites rather than in unidirectional composites, as woven fabric composites offer additional constrain in lateral direction, dimensional stability and enhanced toughness [165]. Therefore, Ng et al. carried out the strength comparison between OHT strength of the specimens with moulded and drilled hole specimens made of 2/2 twill weave T300 carbon/epoxy woven fabric composite. In the case of the drilled hole specimens, the strength predictably decreased with the increasing hole diameter [165]. In the case of the moulded hole specimens, however, the strength tendency was not linear: 499 MPa for 3-mm hole specimen, 453 MPa for 6-mm specimen and 486 MPa for 9-mm hole specimen [165]. The strengths for moulded hole specimens were by 15%, 15% and 46% higher than for the drilled hole specimens, respectively, for the hole dimensions of 3 mm, 6 mm, and 9 mm [165]. The authors attribute the surprising rise of the strength for the specimens with 9 mm moulded holes to the fact that for a specimen with large moulded hole under tension, the curved fibre yarns at notch root have the capability to move towards the centre of the moulded hole and hence the location of maximum stress concentration is at the straight fibre yarn instead of the curved yarn [165]. Besides, the curvature at the hole edge becomes very small for the largest hole and the material at the vicinity can be regarded as an unnotched strip with high fibre volume fraction [165]. Hufenbach et al. investigated the strength of carbon reinforced thermoplastic composite with moulded holes [168]. The manufacturing process of such holes is slightly different for the thermoplastic than for thermoset matrix composites, but the basic idea of arranging reinforcing fibres around pins during moulding remains the same [168]. The authors reported excessive fibre shifting resulting in low volume fraction regions and local thickening of the composite around the holes for the investigated kind of composite [168]. Bearing tests were performed on specimens with drilled and moulded holes with diameters 6 mm, 8 mm and 10 mm. The ultimate strength was higher for all the hole diameters for moulded than for drilled holes. The highest difference, 36%, was measured for 10-mm hole [168]. Lin et al. considered the open-hole failure of drilled and moulded holes in [0,90]_S_, and [±45]_S_ glass/polyester woven-roving composites in tensile test [166]. Experimental results show that the laminate [0,90]_S_ exhibits larger failure strength, smaller initial stiffness and larger failure strain for moulded holes than for drilled holes (Figure 28a). For [±45]_S_ laminates with a moulded hole, the failure strength and initial stiffness show no improvement. Only the strain at failure is improved in this case (Figure 28b) [166].

Lin and Tsai considered the failure of drilled and moulded bolted joints in [0,90]_S_ and [±45]_S_ glass/polyester woven-roving laminates in tensile test [167]. Experimental results show that the specimens with moulded holes exhibit higher joint strength than those with the drilled holes for e/d = 1, whereas, the failure strengths are almost the same for e/d > 2. When e/d = 1, bearing and shear-out failure modes were found in laminates with moulded and drilled hole respectively [167]. Matsuzaki et al. investigated the strength of single lap shear bolted joint of aluminum plate and glass fabric/epoxy composite with moulded holes [169]. The composite was co-cured with the aluminum plate and the bolts. The strength of such specimens was compared with the specimens with the same geometry, but with drilled holes. Static and fatigue tensile tests were performed. In the case of the static test both types of specimen failed by bolt failure demonstrating almost the same ultimate strength, so the advantage of the moulded joint was not evaluated. However, the moulded bolted joints had significantly higher fatigue strength. The fatigue failure mode of the moulded bolted joints was a mixture of bolt failure and bearing failure involving delamination and fibre breakage [169]. The specimens with drilled holes failed by bolt failure, which again disables the opportunity to compare the two types of the specimens impartially.

Moulded holes have not been utilized in commercial applications yet, presumably due to many challenges concerning their manufacturing and maintaining dimensional tolerances. The effect of moulded holes on performance of bolted joints is summarized in Table 5.

## 12. Forged Composites and 3D printing

Quite frequently, bolted connections are applied in joints between movable parts [170,171] where bolt works as an axis of rotation. These movable parts can be made of composite materials so metal bonded inserts are necessary to work as bushings or to install and stabilize the ball bearing or spherical joint. Manufacturing of movable composite parts is usually more complicated than typical parts. Movable parts can be very small and/or tree-dimensional with many details like in the case of the car suspension [6,172,173,174]. Therefore conventional manual lay-up becomes tricky. Necessary installation of metal bonded inserts precisely in right places makes it even more difficult and requires more machining which decreases the strength of the joint. Forged composites technology described in [6] seems to be promising in this case because it not only decreases the time of part manufacturing but also facilitates installation of metal inserts during the composite material lay-up before curing. Simultaneous composite material lay-up with metal inserts positioning before curing allows to avoid drilling or machining of the holes in cured part. Therefore, it allows to obtain quality of the composite material comparable to the moulded hole technique. Metal inserts installation during material lay-up was already performed in the past [170,171]. However, precise positioning of inserts and continuous fibrous reinforcement at the same time was extremely difficult and required very skillful personnel thus making this technique not suitable for mass or even serial production. In the case of forged composites technology the charge consists of prepreg including discontinuous fibrous reinforcement. The charge is put into the mould inaccurately and then fit to the mould with application of very high pressure. If metal inserts are preinstalled in the mould before the charge, then high pressure at the end of process would fit the charge around the inserts as in any other place in the mould. As a result, composite part with metal insert would be manufactured in one step without any need for machining. The only problem occurs in selection of resin which should be good enough for the composite matrix and provide sufficient adhesion to the metal insert. However, if metal insert is equipped with interlocking structures [175,176,177,178,179,180,181,182,183,184], then composite charge would be fit into this interlocking structure creating thus mechanical connection [30] between the insert and the composite part. This mechanical connection can be very strong because shape of the interlocking structures could be very complex thanks to application of 3D printing [184] which is further elaborated in the second part of the current review [30].

## 13. Cost and Installation Challenges

Possible weight reduction is one of the most important reasons why composite materials are applied. Lighter parts and their connections allow to decrease the weight of the final product making it more economical.

In the case of bolted joining, due to the huge number of holes frequently required for the assembly of structural components, hole-making operations must be designed ensuring competitive levels of productivity and cost per operation, while maintaining the required quality of the machined surface [89]. It is reported that the rejection of the composite parts due to drilling-induced delamination damages during the final assembly is as high as 60% [96,98,104], which makes the bolted assembly process ineffective and very cost- and time-consuming. Especially, the economic impact of this is significant considering that the holes are drilled in the final step of the product manufacturing, so the rejections due to poor hole quality are very costly [101,104]. Fortunately the number of rejections can be decreased with application of several methods.

- Proper selection of the drill geometry is perhaps the least expensive method since increase of cost results only from the time necessary to design and manufacture non-standard drills. On the other hand quality of drilling can be significantly increased this way.
- Tool wear leads to the stronger composite damage during drilling [80,85,86,87,88,89] both mechanical and thermal. Application of diamond coated drills increases the life time of the drill by the factor of 8–12 [79,80,81,82,83]. On the other hand, diamond coated drills are obviously expensive, so it has to be decided if increased life-time balances the cost increase for each specific case.
- Lower feed rate and cutting speed of drilling also assure the better hole quality. However, decreasing those parameters means that the machining time would be longer, which has significant economic impact in the cases where large number of holes is necessary.

As can be seen each of these methods complicates the joint-making process and generates additional costs. Therefore, their application has to be carefully optimised.

Moreover, there are several methods increasing strength of the bolted joint for specific holes quality.

- Increased thickness of composite material in the neighbourhood of the joint always allows to increase its strength. However, it also significantly increases the weight reducing advantage of the composite material over metals.
- Compression with washers also allows to increase the strength of the joint but with significantly smaller weight penalty. Unfortunately it is not always possible to use washers. For example the washer cannot be used in the case of bolts with countersunk heads which are used in many aeronautical applications due to aerodynamic reasons. Moreover, the addition of washers doubles the number of joining parts and thus increases the weight and time of assembly.
- Bonded inserts do not mitigate the drilling induced damages, however, the inserts may shield the composite and prevent the damage propagation. On the other hand application of bonded inserts further increases the number of parts and operations to complete composite structure. Weight penalty is moderate—inserts are heavy, but may substitute washers, which are often used anyway.
- Local hybridization with application of titanium foil allows to increase the strength of the joint, however, its influence on the drilling-induced damages is not sufficiently investigated. High complication level results from substitution of single composite plies by titanium foil. On the other hand weight penalty is moderate. It results from greater density of titanium foil over replaced composite plies.
- Fibre steering is another way to reinforce composite-metal bolted joint. Again the influence on the drilling-induced damages is not sufficiently investigated. On the other hand this method is quite complicated because of the need of aligning roving bundles along principal stress lines or load paths. At the same time the weight penalty is also high. Roving bundles often have to be added between each two composite plies doubling the structure weight around the hole.
- Inserting Z-pins or stitching also allows to reinforce the connection but, as in the previous cases, its impact on the drilling-induced damages is not known well enough. On the other hand complication level is high, but weight penalty is moderate since additional reinforcement adds weight, but constitutes low percentage of the structure weight.

Each of these methods complicates the joint-making process and generates additional costs. They also increase the weight of the joint.

Moulded holes technique is a separate case because it eliminates the need to drill holes, and thus eliminates also drilling-induced damages. Moreover, this technique does not increase the weight of the joint. However, complication level is high because special moulds with pins are required and careful aligning the fibres around the pins is necessary.

## 14. Summary

The factors that influence the strength of the bolted joints in composite materials: clamping torque, clearance between the bolt, and the material have been described in the present work. It was found out that the increase in clamping pressure by applying the bolt torque have a positive effect on the bolted joint static and fatigue strength [14,15,25,42,44,45,62]. However, only moderate torques improve the situation significantly, whereas, further increase of it yields virtually no effect or may even lead to the drop of strength [14,15,43,44]. Clearance, which is unavoidable in bolted joints, decreases the contact area between the bolt and the composite. Majority of studies have shown, that it leads to the decrease in static bearing strength of the joints [15,28,46,47]. However, a study concerning the fatigue strength revealed that the fatigue strength may be improved by the presence of clearance [48].

Drilling holes which are necessary to join composite parts by a bolt is a source of problem. Undesirable damages (such as delamination, fibre pull-out and microbuckling [26]) induced by drilling reduce the static and fatigue strength of the joints. Several measures have been invented in order to mitigate the drilling-induced damages in composites. As the drills used for drilling composite materials wear quickly, it is recommended to use diamond-coated or uncoated tools made of cemented carbide as well as polycrystalline diamond drills, which are more resistant to wear than steel drills when drilling composite materials [79,81]. As worn drills cause more damage in composite than new ones [83,89], it is also recommended to replace worn drill bits frequently. The magnitude of delamination caused by drilling can be controlled by the proper selection of drilling parameters [101]. The majority of the cited authors proved that the feed speed/rate and the thrust force, which depends on the feed [101,104], have the highest influence on the delamination magnitude [77,83,98,106,107], while the cutting speed is the parameter that has the highest effect on the hole surface quality [106]. The push-out delamination can be also mitigated by mechanical supports, which backup and thus prevent the separation of the bottom ply [91,97,102,105]. The drill bit geometry is also reported to influence the rise of the damages significantly, however, no clear conclusion, which geometry is the best, can be drawn on the basis of the available literature [93,95,113,114,115].

In the present work, the techniques developed in order to improve the performance of the bolted joints in composite materials are also described. Those techniques include:

- bonding metal inserts around the hole,
- substitution of some composite plies around the holes by titanium foil,
- fibre steering technique which consists in adding roving yarns shaped along principal stress lines or load paths around the holes in composite,
- adding reinforcement through the thickness or in plane of the composite around the holes
- manufacturing moulded holes in composite, which assure the fibre continuity around the holes and prevents drilling-induced damages.

Since all of those techniques have advantages and disadvantages, they were summarised in Table 6. The table lists also the maximum strength increase gained for each technique cited in literature compared to the plain bolted joint.

The analysis of Table 6 shows that two techniques seem particularly promising: titanium foil inserts and moulded holes. The first technique allows to increase the strength of the joint by high value of 158% with moderate weight penalty. On the other hand, this technique complicates the manufacturing process and its influence on the drilling-induced damages is unknown. The moulded hole technique also increases the strength of the joint by lower, but significant value of 46% with no weight penalty. It also prevents the drilling-induced damages by eliminating the drilling process. However, this technique also complicates the manufacturing process.

## 15. Conclusions

The present article is the first part of the literature review concerning the mechanical methods suitable to join composite and metal elements. It focuses on the various aspects of bolted joining and methods developed to improve the performance of bolted joints in composite materials. The presented advantages and disadvantages of those solutions along with the results of the experiments conducted in order to evaluate their usefulness and feasibility should constitute a guide suitable for researchers and engineers looking for novel solutions to enhance the performance of composite-metal and composite-composite bolted joints.

## Figures and Tables

**Table 1:** Summary of metal bonded inserts effect on performance of bolted joints in composite materials.

Drilling-Induced Damages	Complication Level of the Technique	Weight Penalty	Maximum Strength Increase Compared to Plain Joint (Experimental Results)
This technique does not mitigate the drilling induced damages, however, the inserts may shield the composite and thus prevent the damage propagation.	Moderate complication level (bonding inserts to complete composite structure)	Moderate (inserts are heavy, but may substitute washers, which are often used anyway)	The maximum strength of the joint was increased by 24% [18]

**Table 2:** Summary of titanium foil inserts effect on performance of bolted joints in composite materials.

Drilling-Induced Damages	Complication Level of the Technique	Weight Penalty	Maximum Strength Increase Compared to Plain Joint (Experimental Results)
The influence on the drilling-induced damages is not known.	High complication level (substitution of single composite plies by titanium foil)	Moderate (titanium foil is heavier than composite plies)	Tensile strength of a three-row bolted joint was increased by 158% [19]

**Table 3:** Summary of fibre steering effect on performance of bolted joints in composite materials.

Drilling-Induced Damages	Complication Level of the Technique	Weight Penalty	Maximum Strength Increase Compared to Plain Joint (Experimental Results)
The influence on the drilling-induced damages is not known.	High complication level (Aligning roving bundles along principal stress lines or load paths)	High (roving bundles often have to be added between each two composite plies doubling the structure weight around the hole)	Bearing strength was increased by 36% for the modified steered pattern [40]

**Table 4:** Summary of additional reinforcement effect on performance of bolted joints in composite materials.

Drilling-Induced Damages	Complication Level of the Technique	Weight Penalty	Maximum Strength Increase Compared to Plain Joint (Experimental Results)
The influence on the drilling-induced damages is not known.	High complication level (inserting Z-pins or stitching)	Moderate (additional reinforcement is an added weight, but constitutes low percentage of the structure weight)	Z-pins improved the bearing failure strength of composite joint by 7.4–9.8 % [156]

**Table 5:** Summary of moulded holes effect on performance of bolted joints in composite materials.

Drilling-Induced Damages	Complication Level of the Technique	Weight Penalty	Maximum Strength Increase Compared to Plain Joint (Experimental Results)
The technique eliminates the need to drill holes, thus eliminates also drilling-induced damages.	High complication level (manufacturing of special moulds with pins, aligning fibres around the pins)	None	The strengths for moulded hole specimens were by 46% higher than for the drilled hole specimens [165]

**Table 6:** Summary of techniques used to improve performance of bolted joints in composite materials.

	Technique				
	Bonded Inserts	Titanium Foil	Fibre Steering	Additional Reinforcement	Moulded Holes
Drilling-Induced Damages	This technique does not mitigate the drilling induced damages, however, the inserts may shield the composite and thus prevent the damage propagation.	The influence on the drilling-induced damages is not known.	The influence on the drilling-induced damages is not known.	The influence on the drilling-induced damages is not known.	The technique eliminates the need to drill holes, thus eliminates also drilling-induced damages.
Complication Level of the Technique	Moderate complication level (bonding inserts to complete composite structure)	High complication level (substitution of single composite plies by titanium foil)	High complication level (Aligning roving bundles along principal stress lines or load paths)	High complication level (inserting Z-pins or stitching)	High complication level (manufacturing of special moulds with pins, aligning fibres around the pins)
Weight Penalty	Moderate (inserts are heavy, but may substitute washers, which are often used anyway)	Moderate (titanium foil is heavier than composite plies)	High (roving bundles often have to be added between each two composite plies doubling the structure weight around the hole)	Moderate (additional reinforcement is an added weight, but constitutes low percentage of the structure weight)	None
Maximum Strength Increase Compared to Plain Joint (Experimental Results)	The maximum strength of the joint was increased by 24% [18]	Tensile strength of a three-row bolted joint was increased by 158% [19]	Bearing strength was increased by 36% for the modified steered pattern [40]	Z-pins improved the bearing failure strength of composite joint by 7.4–9.8 % [156]	The strengths for moulded hole specimens were by 46% higher than for the drilled hole specimens [165]
